# Viral hijacking of a replicative helicase loader and its implications for helicase loading control and phage replication

**DOI:** 10.7554/eLife.14158

**Published:** 2016-05-31

**Authors:** Iris V Hood, James M Berger

**Affiliations:** 1 Department of Molecular and Cell Biology, California Institute for Quantitative Biosciences, University of California, Berkeley, Berkeley, United States; 2 Department of Biophysics and Biophysical Chemistry, Johns Hopkins University School of Medicine, Baltimore, United States; University of California, Davis, United States

**Keywords:** helicase, helicase loader, AAA+ ATPase, bacteriophage, replication, Virus, *S. aureus*

## Abstract

Replisome assembly requires the loading of replicative hexameric helicases onto origins by AAA+ ATPases. How loader activity is appropriately controlled remains unclear. Here, we use structural and biochemical analyses to establish how an antimicrobial phage protein interferes with the function of the *Staphylococcus aureus* replicative helicase loader, DnaI. The viral protein binds to the loader’s AAA+ ATPase domain, allowing binding of the host replicative helicase but impeding loader self-assembly and ATPase activity. Close inspection of the complex highlights an unexpected locus for the binding of an interdomain linker element in DnaI/DnaC-family proteins. We find that the inhibitor protein is genetically coupled to a phage-encoded homolog of the bacterial helicase loader, which we show binds to the host helicase but not to the inhibitor itself. These findings establish a new approach by which viruses can hijack host replication processes and explain how loader activity is internally regulated to prevent aberrant auto-association.

**DOI:** http://dx.doi.org/10.7554/eLife.14158.001

## eLife digest

Cells must copy their DNA in order to grow and divide. DNA replication begins when a small region of the DNA double helix is unwound to expose single strands of DNA. A protein called a helicase is then shepherded onto the unwound DNA regions by other proteins known as loaders. Once loaded, the helicase can unwind long stretches of the chromosome in which the DNA is packaged, producing the template required by the replication machinery to duplicate the DNA. This process must be accurately executed to avoid generating errors that could damage the DNA and potentially cause cells to die.

DnaI is a helicase loader protein that is found in some types of bacteria. In the disease-causing bacterial species *Staphylococcus aureus (S. aureus*), an inhibitor protein from a virus that infects the bacteria can interact with DnaI and halt *S. aureus* DNA replication, leading to cell death. However, it has not been understood how this viral protein controls the activity of the loader molecules.

DnaI consists of three regions: one that binds to the helicase, a short 'linker' region, and a third element that harnesses chemical energy (in the form of a small high-energy molecule called ATP) to drive the loader’s activity. Using biochemical and structural techniques, Hood and Berger now show that the viral inhibitor protein interacts with the DnaI loader from *S. aureus* by binding to the loader's ATP-binding region. When the two proteins are bound together, the loader can still bind to its target helicase but it cannot interact with other loader molecules. This defect prevents the loaders from self-assembling into a structure that is required for them to load the replicative helicase.

Hood and Berger also found that the region of DnaI targeted by the inhibitor is important for normally ensuring that the loader molecules self-assemble at the correct place and time. A second unexpected discovery was that the virus encodes its own helicase loader, which binds to the bacterial helicase but not to the viral inhibitor protein. The next stage of work will be to determine whether the regions on the helicase loader that are targeted by the inhibitor and that are important for regulating self-assembly can be selectively disrupted by small molecules to interfere with DNA replication in bacteria.

**DOI:** http://dx.doi.org/10.7554/eLife.14158.002

## Introduction

All cells face the challenging task of copying and passing on genetic information to progeny in an error-free manner as possible (Fuchs and Fujii, 2013 ; Sutera and Lovett, 2006). DNA synthesis is carried out by large, multi-subunit assemblies, termed replisomes, which are assembled at replication origins in accord with cell cycle cues. Dedicated proteins known as initiators play an essential role in selecting replication start sites, remodeling origin DNA, and providing an appropriate platform for recruiting replicative helicases to origins prior to the onset of strand synthesis (Costa et al., 2013; Dutta and Bell, 1997; Kaguni, 2011; Leonard and Grimwade, 2011). Although specific helicase loading mechanisms vary across the three domains of life – archaea, bacteria, and eukaryotes – all appear to rely on replication initiation factors belonging to the AAA+ (ATPases Associated with various cellular Activities) superfamily of nucleotide hydrolases.

**Figure 1.**
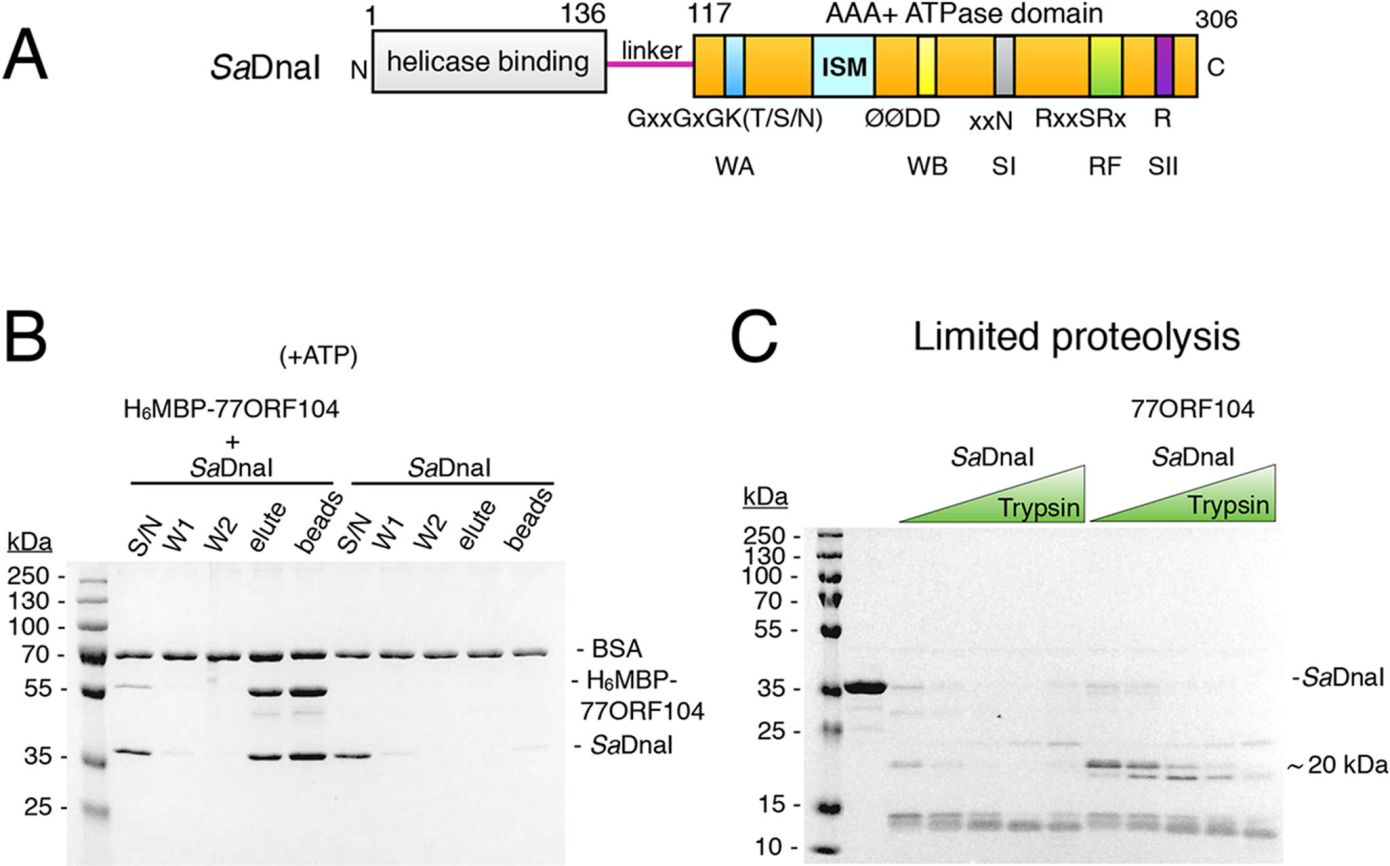
The phage 77 ORF104 protein binds the *S. aureus* DnaI ATPase domain. (**A**) Domain organization of *Sa*DnaI. The N-terminal helicase-binding domain is colored gray, the linker region is magenta, and the AAA+ domain is orange. Numbers refer to amino acid positions. AAA+ ATPase motifs are labeled: Walker-A (WA), Walker-B (WB), Sensor-I (SI), Box VII, Sensor-II (SII), and the initiator/loader specific motif (ISM). (**B**) SDS-PAGE analysis of amylose pull-downs between His_6_MBP-77ORF104 and full-length *Sa*DnaI (with ATP). A DnaI alone control is shown. Supernatant – S/N, W1 and W2 – washes. (**C**) Limited trypsin proteolysis and SDS-PAGE analysis of full-length *Sa*DnaI in the presence of 77ORF104. A ~20kDa band of *Sa*DnaI is stabilized in the presence of 77ORF104. **DOI:** http://dx.doi.org/10.7554/eLife.14158.003

The timely and accurate deposition of replicative helicases onto origin DNA is a highly coordinated and regulated process. In bacteria, replication initiation relies on the DnaA initiator, which recognizes and marks the bacterial replication origin (Bramhill and Kornberg, 1988a; 1988b; Funnell et al., 1987; Hsu et al., 1994). During initiation, DnaA actively opens an AT-rich region of the origin (Bramhill and Kornberg, 1988a, 1988b; Dixon and Kornberg, 1984; Funnell et al., 1987; Gille and Messer, 1991; Hsu et al., 1994; Skarstad et al., 1990), termed a DNA-unwinding element (DUE) (Kowalski and Eddy, 1989), and helps to recruit two copies of the replicative helicase to the newly melted single strands. In certain Gram-negative bacteria, a protein known as DnaC assists with loading of the helicase (known in these organisms as DnaB); many Gram-positive species retain a homolog of DnaC termed DnaI. Both DnaC and DnaI are composed of an N-terminal helicase binding domain that connects to a C-terminal AAA+ ATPase domain by a variable linker region of unknown function (Loscha et al., 2009) (Figure 1A). There is evidence that ATPase activity by DnaC/I proteins controls key aspects of the helicase loading cycle and may be auto-regulated (Davey et al., 2002; Ioannou et al., 2006; Learn et al., 1997); however, the mechanism by which this control is exerted is not understood.

Given the marked rise in multi-drug resistant bacterial strains, along with associated human fatalities (Klein et al., 2007; 2013; Salgado et al., 2003), there is renewed interest in exploring bacteriophage genomes to discover new antimicrobial agents. Although bacterial DNA replication proteins are strikingly distinct from those found in eukaryotes and archaea (Leipe et al., 1999), antibiotics currently on the market do not target replication components directly, suggesting that this system may be a valuable prospective drug target (McHenry, 2011; Robinson et al., 2010; 2012). Along these lines, a small, 52 amino acid protein from phage strain 77, encoded by the *ORF104* gene, has been reported to interact directly with the *Staphylococcus aureus* helicase loader, DnaI, and to inhibit host DNA replication in vivo (Liu et al., 2004). The discovery of 77ORF104 marks the first known instance in which the replication initiation machinery of a bacterium can be inhibited by an exogenous factor (Liu et al., 2004); however, where the inhibitor protein associates with the helicase loader and how it represses helicase loader activity has remained unknown.

To gain insights into both bacterial helicase loader mechanisms and how they may be disrupted by an exogenous viral system, we used a combination of structural, biochemical, and comparative studies to define how the phage 77 ORF104 protein inhibits *S. aureus* DnaI (*Sa*DnaI). We show that the phage protein binds directly to DnaI’s AAA+ ATPase domain, where it both remodels a region critical for loader self-assembly and sterically masks a known loader-loader interaction site. Inhibitor binding, which represses the helicase loader’s ATP hydrolysis activity, is found to exploit a surface region normally occupied by a portion of the linker region that connects the N- and C-terminal domains of DnaI. Together, our data not only establish a new means by which viruses can inhibit DNA replication, but also indicate that bacterial helicase loaders possess an unanticipated auto-regulatory element, located within their variable linker region, that serves to help restrict premature loader self-assembly.

## Results

### The phage 77 ORF104 protein binds the SaDnaI C-terminal AAA+ domain in a nucleotide-independent manner

To begin to probe the interaction between 77ORF104 and the helicase loader from *S. aureus (Sa*DnaI), we first cloned, expressed, and purified both full-length proteins. By performing amylose pull-down assays using the tagged inhibitor protein as bait and DnaI as prey (Figure 1B), we confirmed that 77ORF104 associates with *Sa*DnaI as previously reported (Liu et al., 2004). We then conducted limited proteolysis studies of *Sa*DnaI in both the presence and absence of 77ORF104 to determine where the phage protein might bind. Inspection of the reactions using SDS-PAGE showed relatively rapid degradation of *Sa*DnaI when the phage protein was omitted. By contrast, when 77ORF104 was added to the full-length loader, a distinct 20kDa species remained protected from digestion (Figure 1C).

**Figure 2.**
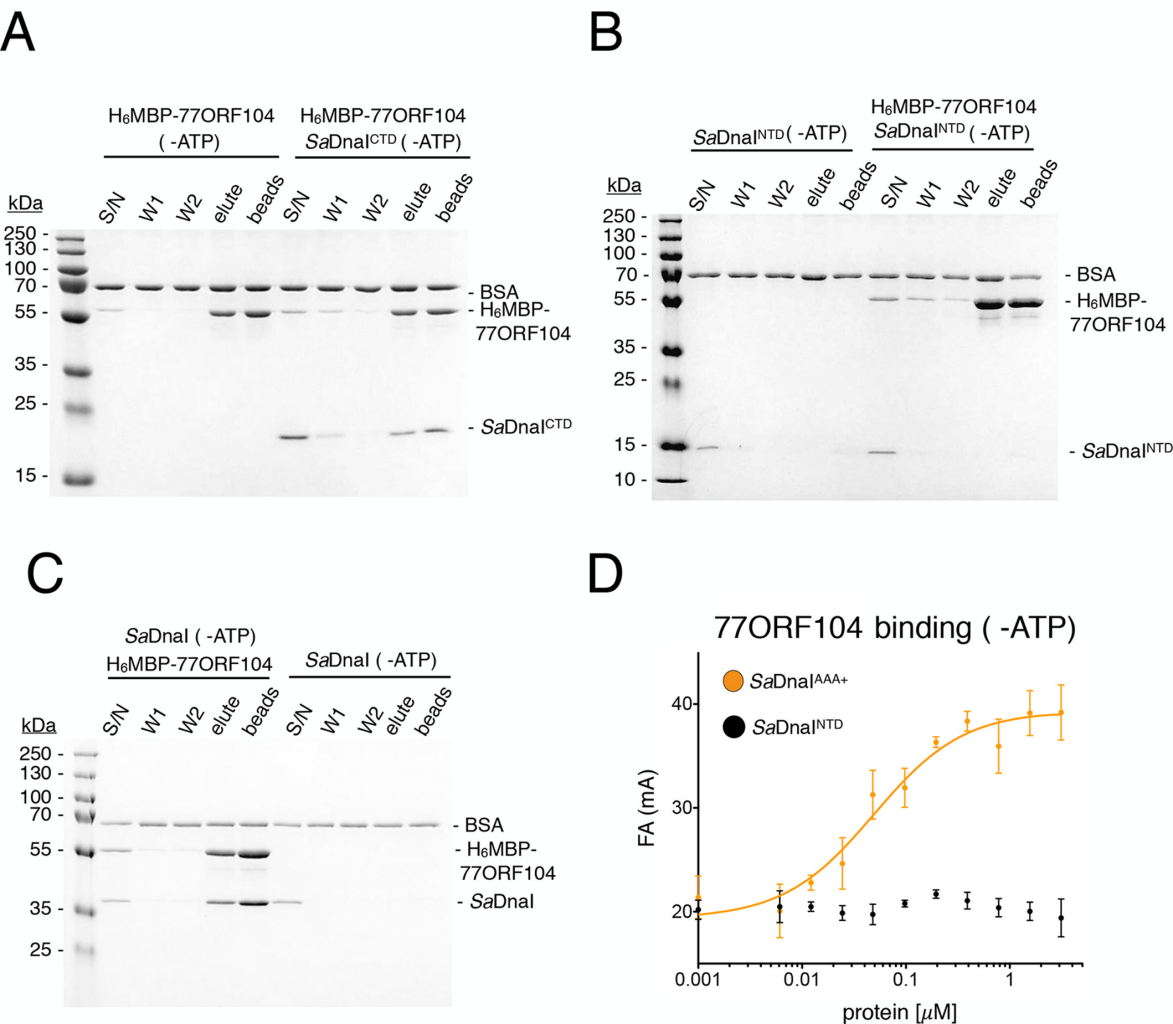
Binding of 77ORF104 to the *Sa*DnaI AAA+ domain is nucleotide-independent. SDS-PAGE analysis of amylose pull-downs between His_6_MBP-77ORF104 and (**A**) *Sa*DnaI^CTD^, (**B**) *Sa*DnaI^NTD^, or (**C**) full-length *Sa*DnaI in the absence of ATP. DnaI and 77ORF104 alone controls are also shown. (**D**) Binding the phage 77 ORF104 protein to *Sa*DnaI^AAA+^ and *Sa*DnaI^NTD^ as measured by a change in fluorescence anisotropy (ΔFA – change in milli-anisotropy units). The X-axis denotes protein concentration. Data points and error bars derive from three-independent experiments. No measurable binding was observed for *Sa*DnaI^NTD^. **DOI:** http://dx.doi.org/10.7554/eLife.14158.004

Having established that 77ORF104 appears to bind to and/or mask a defined portion of *Sa*DnaI, we next sought to define the interacting regions more precisely. We therefore expressed and purified individual domains of *Sa*DnaI, including the N-terminal domain (NTD) and linker, the AAA+ domain and linker (CTD), and the AAA+ domain alone. Turning again to pull-down assays with tagged 77ORF104, we found that the phage protein was capable of retaining both the isolated C-terminal AAA+ ATPase domain (with or without the preceding linker region) but not the N-terminal domain on its own (Figure 2A–B). Given this finding, we next tested whether nucleotide was required for *Sa*DnaI’s association with 77ORF104; ATP was found to be dispensable for association with the full-length loader (compare Figures 1B, 2C). Together, these findings establish that the phage inhibitor binds directly to the ATPase fold of DnaI but that this interaction is independent of nucleotide.

### Structure of a ADP•BeF_3_-bound SaDnaI^AAA+^•77ORF104 complex

**Figure 3.**
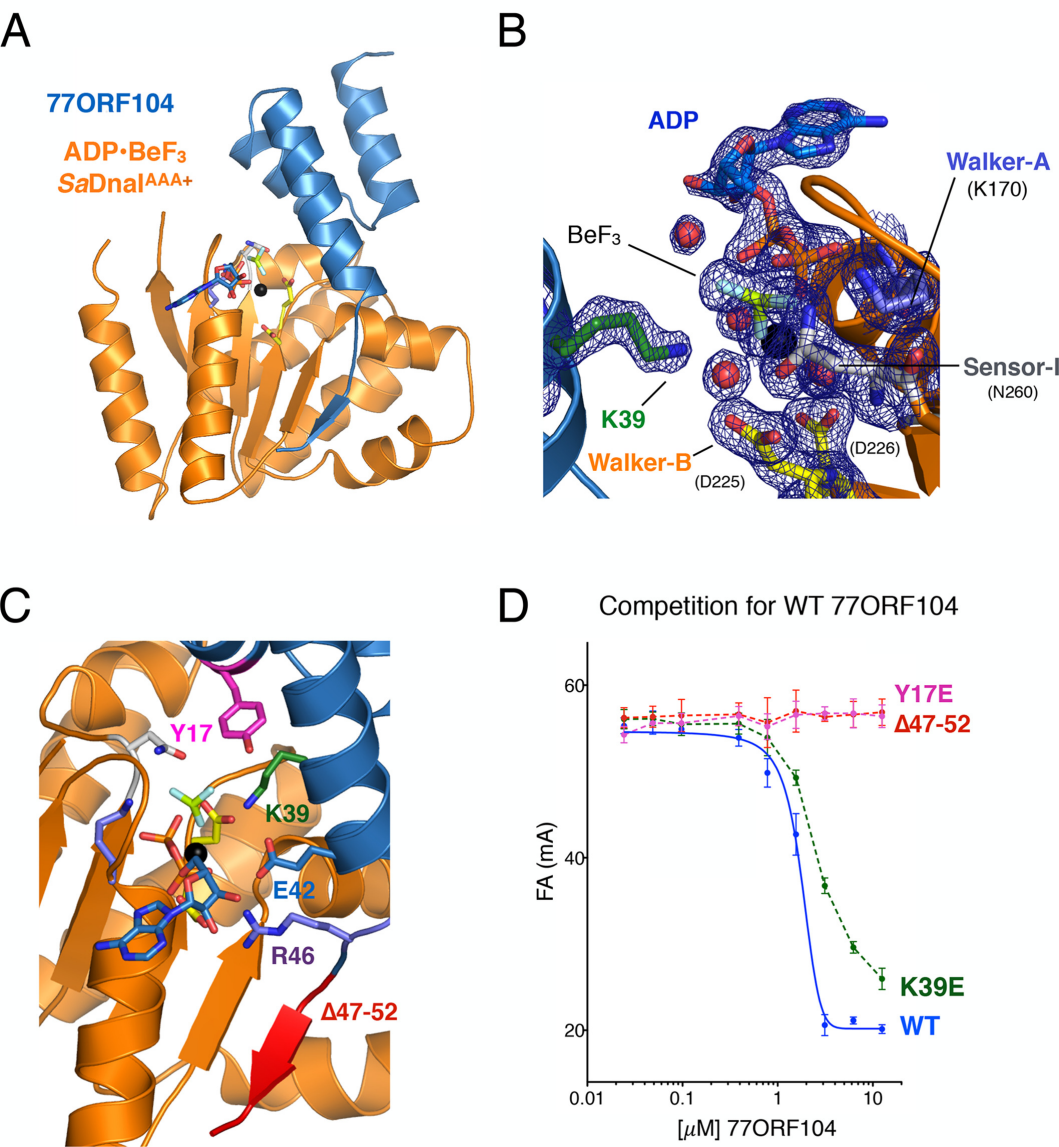
Structure of the ADP•BeF_3_-bound *Sa*DnaI^AAA+^•77ORF104 complex and biochemical validation of observed interactions. (**A**) Overall structure of ADP•BeF_3_-bound *Sa*DnaI^AAA+^ complexed with 77ORF104. *Sa*DnaI^AAA+^ is colored orange and 77ORF104 sky-blue. ADP (dark blue), BeF_3_ (limon-teal) and a magnesium ion (black), are shown within the ATP binding site of *Sa*DnaI^AAA+^. (**B**) Close-up view of the *Sa*DnaI ^AAA+^ ATP binding site. Conserved motifs are colored: Walker-A (Lys170) (blue), Walker-B (Asp225 and Asp226) (yellow), and Sensor-I (Asn260) (grey). Lys39 from 77ORF104 is colored green, a magnesium ion in black and liganding waters in red. Refined 2Fo-Fc electron density is shown for a portion of the region, contoured at 1.6 σ. (**C**) Analysis of the ADP•BeF_3_-bound *Sa*DnaI^AAA+^•77ORF104 interface. Several residues from 77ORF104 that participate in the interaction are shown as sticks and labeled. Three elements selected for mutagenesis studies are colored pink (Tyr17), green (Lys39), and red (residues 47–52). (**D**) Competition assay showing the ability of different 77ORF104 mutants to compete away wild-type, N-terminally labeled 77ORF104 from interacting with *Sa*DnaI^AAA+^. Competition is evident by a decrease in fluorescence anisotropy (ΔFA – change in milli-anisotropy units) as labeled protein is displaced by the unlabeled 77ORF104 competitor. **DOI:** http://dx.doi.org/10.7554/eLife.14158.005

To better understand the physical basis for the phage 77 ORF104-*Sa*DnaI interaction, we set out to determine a crystal structure of the complex. To define a minimal helicase loader construct for crystallization screening, we fluorescently labeled the N-terminus of wild-type 77ORF104 (Materials and **methods**) and performed fluorescence anisotropy-based binding assays with various purified *Sa*DnaI truncations. In accord with our pull-down studies, the core AAA+ domain of *Sa*DnaI^AAA+^ proved sufficient for binding to 77ORF104, while the N-terminal domain of *Sa*DnaI showed no evidence of binding (Figure 2D). The calculated apparent K_d_ (K_d, app_ = 49 nM ± 19 nM) for the inhibitor-loader AAA+ domain interaction proved comparable to that previously reported for the 77ORF104 protein and full-length *Sa*DnaI (K_d_ = ~50 nM as reported by surface plasmon resonance) (Liu et al., 2004). Together, these data show that only the core AAA+ domain of the *S. aureus* helicase loader is required for high affinity association with the phage inhibitor protein, and suggested that the *Sa*DnaI ATPase fold might serve as a promising candidate for co-crystallization studies. Following screening, the successful acquisition of crystals, and data collection (see Materials and methods), we determined a crystal structure of the AAA+ domain of *Sa*DnaI bound to both 77ORF104 and an ATP-mimetic, ADP•BeF_3_ (Figure 3A), using single-wavelength anomalous dispersion for phasing. Following several rounds of building and refinement, the model converged at an R_work_/R_free_ of 18.0%/21.8% for the resolution range of 47.4–1.9 Å (Table 1). The final model contains residues 136–300 for *Sa*DnaI and all 52 residues of 77ORF104, and displays good overall stereochemistry.

**Table 1.** Data collection, phasing and refinement statistics for apo SaDnaI^AAA+^ and ADP*•*BeF_3_-*Sa*DnaI^AAA+^*•*77ORF104 structures. **DOI:** http://dx.doi.org/10.7554/eLife.14158.006

Construct:	'apo' *Sa*DnaI^AAA+^	77ORF104-*Sa*DnaI^AAA+^
**Data Collection**		
Beamline	BNL NSLS X25	BNL NSLS X25
Wavelength	1.500	0.979
Space group	P2_1_2_1_2_1_	P6_5_22
Cell edges (Å)	113.10, 126.26, 183.34	73.17, 73.17, 189.72
Cell angles (°)	90.0, 90.0, 90.0	90.0, 90.0, 120.0
Resolution range (Å)	48.1–2.6 (2.74– 2.6)	44.8–1.9 (1.97–1.9)*^a^*
Unique reflections	81,324 (11,704)	24,589 (2,390)
Completeness (%)	99.9 (99.5)	100.00 (100.00)
R_merge_	0.16 (4.00)	0.165 (1.55)
R_meas_	0.17 (4.33)	0.17 (1.65)
R_pim_	0.066 (1.66)	.054 (.57)
Redundancy	13.3 (13.2)	18.5 (15.7)
I/σ(I)	3.4 (0.2)	14.82 (1.44)
Wilson B-Factor	29.6	26.6
CC 1/2	0.996 (0.684)	0.999 (0.693)
**Phasing**		
# of sites	NA	4
FOM^b^	NA	0.90 (0.54)
**Refinement**		
Resolution limits (Å)	48.13 (2.6)	47.4 (1.9)
*R_work_*(*R_free_*)^b^	22.7 (26.5)	18.0 (21.8)
No. protein residues	1975	223
No. solvent/ligand molecules	67/9	209/32
RMSD Bond, Å	0.003	0.012
RMSD Angle, °	0.540	1.335
**Protein geometry**		
Ramachandran preferred/outliers (%)	95.87/0.61	99.5/0
Rotamer outliers (%)	0.48	0

^a^Numbers in parentheses refer to the highest resolution shell.

^b^Five percent of the total number of reflections were used to calculate R_free_.

Examination of the ADP•BeF3-bound *Sa*DnaI^AAA+^•77ORF104 complex revealed that the phage protein binds to the AAA+ domain of *Sa*DnaI in a 1:1 manner (Figure 3A). *Sa*DnaI’s core AAA+ domain forms an αβα fold typical of the superfamily, and possesses all of the canonical motifs involved in nucleotide binding, such as the Walker-A, Walker-B and Sensor-I elements (Walker et al., 1982) (Figure 1A). Collectively, these motifs adopt a configuration similar to that seen in structures of other nucleotide-bound AAA+ ATPases (e.g., see [Erzberger and Berger, 2006]), except that the last six C-terminal residues of the structure, which include the Sensor-II amino acid (Arg304), were unresolved (Figure 3B). Inspection of the electron density in the active site revealed clear density for nucleotide binding, permitting modeling of ADP, BeF_3_, and a single Mg^2+^ ion and its associated waters. Interestingly, 77ORF104 can be seen to directly engage the bound nucleotide. In this regard, Lys39 of the phage inhibitor protein makes one of the more notable contacts, projecting into the *Sa*DnaI^AAA+^ active site to engage the BeF_3_ moiety directly (Figure 3B).

### Structurally observed interactions are important for loader-inhibitor interactions

Upon inspection of the 77ORF104*•Sa*DnaI^AAA+^ binding interface, it became evident that the interaction of these proteins can be divided into roughly three 'hotspots' (Figure 3C). One such locus involves the five most C-terminal residues of the protein, which form a β-strand that associates laterally with one edge of the β-sheet in *Sa*DnaI’s AAA+ domain core. The other two loci involve residues such as Tyr17 and Lys39, which make contacts to or around the nucleotide-binding site of *Sa*DnaI and the associated loader-inhibitor interaction surface.

To determine whether specific contacts observed in the *Sa*DnaI^AAA+^*•*77ORF104 complex are important for the inhibitor’s association with DnaI, we designed, cloned and purified several 77ORF104 mutants based on the structure and tested them for binding to *Sa*DnaI^AAA+^ (Figure 3D). Lys39 and Tyr17 were each mutated to glutamate in the full-length 77ORF104 protein, and the last six amino acids of the inhibitor’s C-terminus were also truncated (77ORF104^Δ47–52^). We then developed a competition-based fluorescence anisotropy assay to assess the ability of different mutant proteins to displace fluorescently labeled, wild-type 77ORF104 from the *Sa*DnaI AAA+ domain. As expected, native 77ORF104 proved capable of competing away the dye-labeled 77ORF104 protein from binding to *Sa*DnaI^AAA+^ (K_i,app_=1.625 μM ± 0.07 μM) (Figure 3D), establishing the utility of the assay. Testing of the 77ORF104^K39E^ mutant revealed only a modest reduction in potency relative to the wild-type protein, indicating that this amino acid serves a relatively peripheral role in stabilizing the inhibitor-loader interface (Figure 3D). By comparison, removal of the C-terminal tail of 77ORF104 or mutation of Tyr17 to glutamate completely abrogated the ability of 77ORF104 to compete for binding by the labeled inhibitor protein (Figure 3D). Together, these data corroborate the structural interactions seen in the 77ORF104•*Sa*DnaI^AAA+^ complex, demonstrating that both Tyr17 and the last five residues of the C-terminal tail of 77ORF104 are particularly critical for the activity of the phage inhibitor. The results with the 77ORF104^K39E^ mutant additionally are consistent with our pull-down studies, which show that the 77ORF104-*Sa*DnaI interaction does not require nucleotide for stable association (Figure 2A–B).

### 77ORF104 blocks DnaI self-assembly by two mechanisms

**Figure 4.**
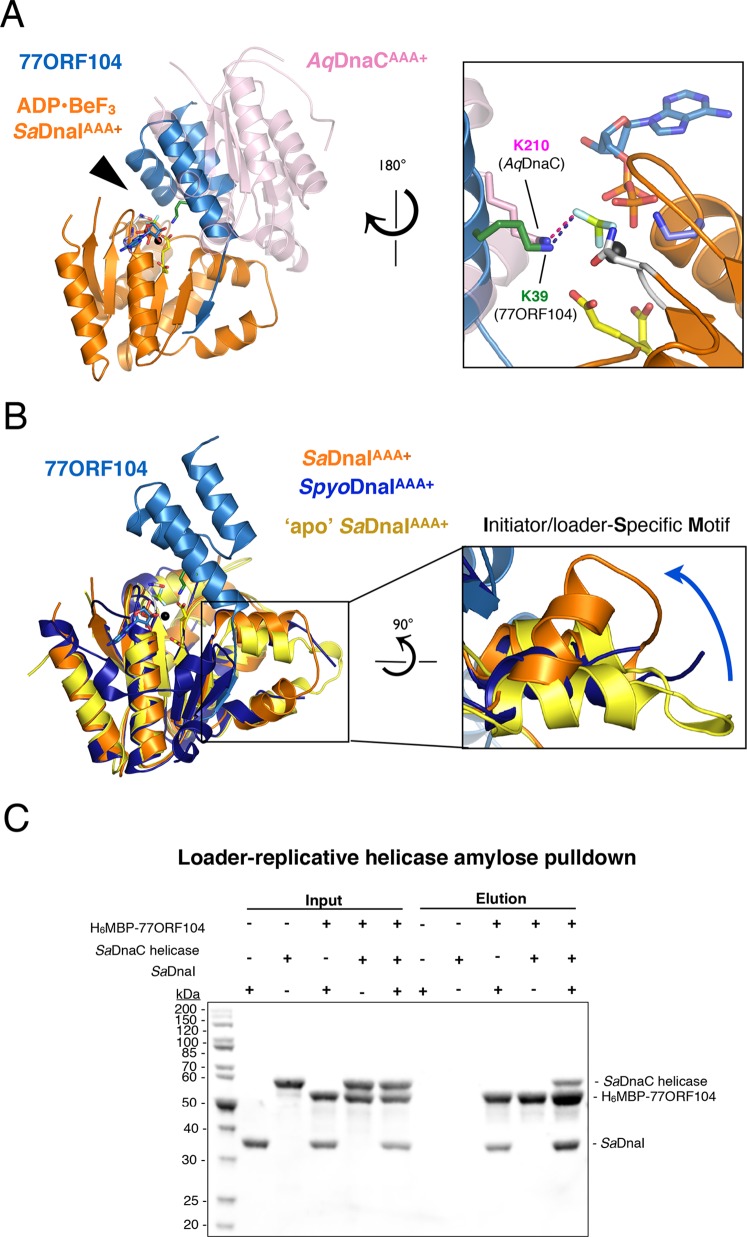
77ORF104 alters the local conformation of the *Sa*DnaI ISM but does not block interactions with the *Sa*DnaC replicative helicase. (**A**) Superposition of a nucleotide-stabilized *Aquifex aeolicus* DnaC^AAA+^ (*Aq*DnaC^AAA+^) dimer (PDB ID 3ECC, [Mott et al., 2008]) onto the *Sa*DnaI^AAA+^•77ORF104 structure. The docking results in a steric clash between 77ORF104 and the neighboring protomer of the *Aq*DnaC^AAA+^ dimer. The inset (rotated 180˚) shows a close-up view of the ATP binding center, highlighting how the dimer-partner of the *Aquifex aeolicus* DnaC^AAA+^ domain projects a positive amino acid (Lys210) into the AAA+ active site in a manner similar that seen for Lys39 from 77ORF104. (**B**) Induction of a local conformational change to the *Sa*DnaI ISM by 77ORF104 can be seen in a comparative structural analysis with other DnaI homologs. The inset (rotated 90˚) shows a close-up view of ISM helices in the 77ORF104-inhibited complex aligned to apo *Sa*DnaI^AAA+^ and *Streptococcus pyogenes* DnaI (*Spyo*DnaI^AAA+^) (PDB ID 2QGZ, Seetharaman *et al*., to be published). The blue arrow indicates the direction of ISM bending by the phage inhibitor protein. (**C**) 77ORF104 associates with *Sa*DnaI when the helicase loader is bound to the host *Sa*DnaC helicase. SDS-PAGE analysis of amylose pull-down experiments using purified His_6_MBP-tagged 77ORF104, *Sa*DnaI, and the *Sa*DnaC replicative helicase. The positions of each protein are indicated on the right; inputs are shown on the left half of the gel. His_6_MBP-tagged 77ORF104 co-precipitates with *Sa*DnaI alone and *Sa*DnaI bound to the *Sa*DnaC replicative helicase, but not with the *Sa*DnaC helicase alone. Pull-down experiments were performed in the absence of nucleotide. Performing the experiment in the presence of nucleotide generated the same result (not shown), indicating nucleotide is not required for *Sa*DnaI to associate with *Sa*DnaC. **DOI:** http://dx.doi.org/10.7554/eLife.14158.007

Having established how the phage inhibitor engages *Sa*DnaI, we sought next to determine how 77ORF104 binding might interfere with specific loader functions. Most AAA+ ATPase systems oligomerize by inter-ATPase domain interactions that juxtapose the active site of one subunit with a catalytically important basic amino acid (the arginine finger) of another subunit (Wendler et al., 2012). In the case of 77ORF104, the protein associates with the nucleotide-binding face of *Sa*DnaI; superposition of the *Sa*DnaI AAA+ domain in our complex onto the AAA+ domain of a single subunit from a nucleotide-assembled dimer of *Aquifex aeolicus* DnaC (Mott et al., 2008) shows that the 77ORF104 protein occupies the same location as that of the neighboring protomer (Figure 4A). This arrangement indicates that the phage inhibitor blocks *Sa*DnaI activity by sterically blocking loader self-assembly.

**Figure 4—figure supplement 1.**
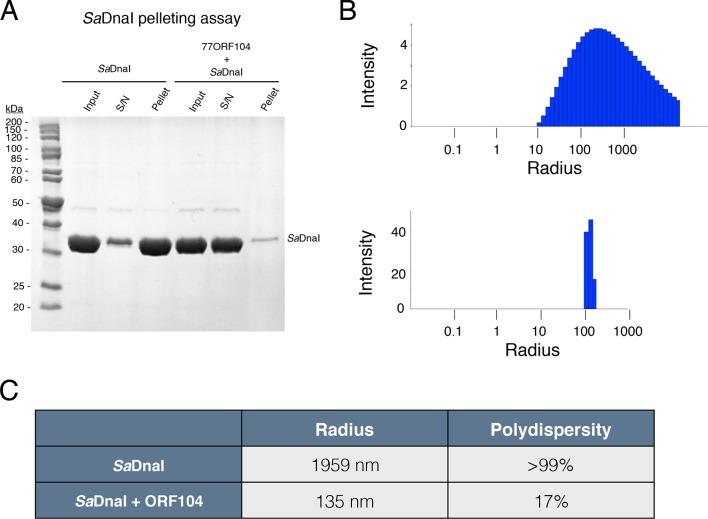
77ORF104 directly interferes with the self-association of *Sa*DnaI. (**A**) SDS-PAGE analysis of centrifugal *Sa*DnaI assembly assay. Lanes from reaction containing *Sa*DnaI with or without 77ORF104 are shown and labeled. S/N – supernatant. (**B**) Dynamic light scattering plots of *Sa*DnaI alone (upper) and *Sa*DnaI in the presence of equimolar 77ORF104 (lower). (**C**) Table representing some the physical parameters calculated from DLS measurements of DnaI alone and in the presence of the ORF104 protein. **DOI:** http://dx.doi.org/10.7554/eLife.14158.008

**Figure 4—figure supplement 2.**
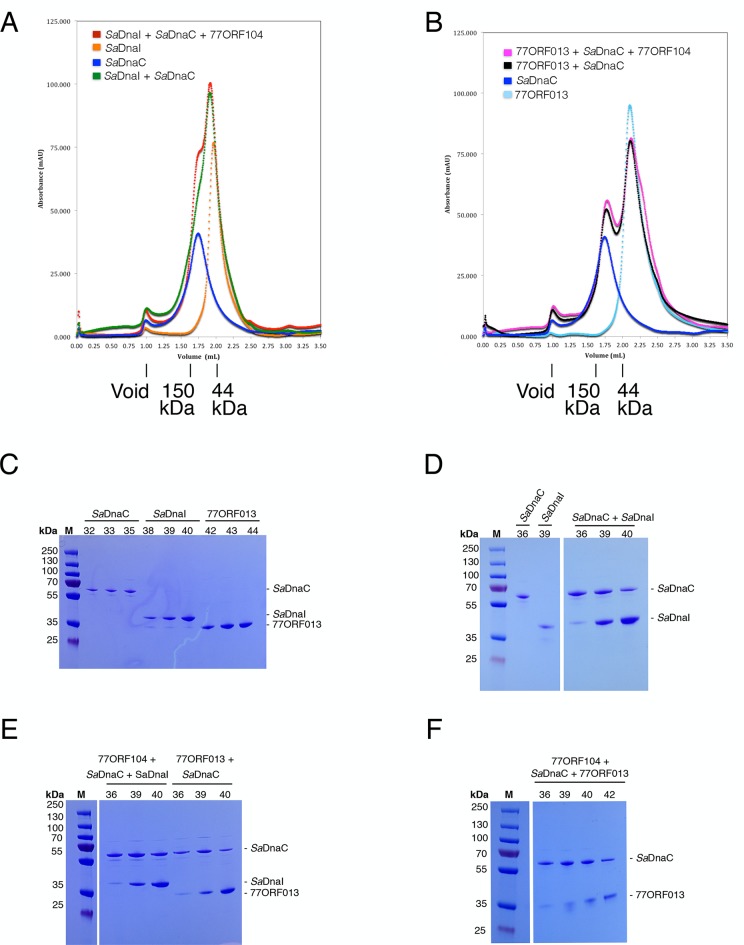
Analytical gel filtration results of *Sa*DnaC, *Sa*DnaI, 77ORF013 and 77ORF104, both alone and in various combinations. The relative positions of marker proteins are indicated. (**A**) Analytical gel filtration chromatographs of *Sa*DnaC and *Sa*DnaI alone, *Sa*DnaC and *Sa*DnaI together, and *Sa*DnaC and *Sa*DnaI with 77ORF104. (**B**) Analytical gel filtration chromatographs of *Sa*DnaC and 77ORF013 alone, *Sa*DnaC and 77ORF013 together, and *Sa*DnaC and 77ORF013 with 77ORF104. (**C**) SDS-PAGE analysis of selected peak fractions for *Sa*DnaC, *Sa*DnaI and 77ORF013 alone. (**D**) SDS-PAGE analysis of selected peak fractions for *Sa*DnaC, *Sa*DnaI and *Sa*DnaC + *Sa*DnaI. (**E**) SDS-PAGE analysis of selected peak fractions for 77ORF104 + *Sa*DnaC + *Sa*DnaI and 77ORF013 + *Sa*DnaC. (**F**) SDS-PAGE analysis of selected peak fractions for 77ORF104 + *Sa*DnaC + 77ORF013. **DOI:** http://dx.doi.org/10.7554/eLife.14158.009

Within AAA+ ATPases, one specialized feature that phylogenetically distinguishes helicase loaders such as DnaI and bacterial replication initiator proteins from other superfamily members is the existence of an extra α-helix that is inserted into one edge of the core AAA+ fold (Iyer et al., 2004). This element, termed the **I**nitiator/loader-**S**pecific **M**otif (ISM) (Dueber et al., 2007), has been shown to be important for self-assembly and function in replicative helicase loaders and initiators (Mott et al., 2008; Dueber et al., 2007; Erzberger et al., 2006; Duderstadt et al., 2011). In nucleotide-oligomerized DnaA and DnaC/I structures, the ISM introduces a significant out-of-plane displacement of adjacent ATPase folds, and appears responsible for pushing these assemblies into a helical, as opposed to closed-ring, formation. As anticipated, based on structural alignments with DnaC/I homologs, the ISM of 77ORF104-bound *Sa*DnaI^AAA+^ forms a V-shaped projection from the central ATPase domain. Inspection of this region, however, revealed that one of the α-helices of the *Sa*DnaI ISM is bent compared to the ISM region of other helicase loaders (Figure 4B). Examination of the crystal contacts in our *Sa*DnaI^AAA+^*•*77ORF104 structure indicates that the change in conformation of the ISM is not the result of packing interactions, indicating that binding of the 77ORF104 inhibitor is responsible for introducing this conformational change in *Sa*DnaI^AAA+^.

To determine whether the conformational change seen in the *Sa*DnaI ISM corresponds to a natural state of *S. aureus* protein, or as a result of binding to phage 77 ORF104, we crystallized and determined the structure of the *Sa*DnaI AAA+ domain in absence of the inhibitor protein (Figure 4B). Structural alignment of the apo and 77ORF104-bound *Sa*DnaI^AAA+^ models shows that the ISM is straight in the absence of the inhibitor, as seen in other helicase loader structures, such as *Streptococcus pyogenes* DnaI (shown in Figure 4B). This result indicates that the bent conformational change visualized for the *Sa*DnaI ISM in the presence of the inhibitor is a consequence of 77ORF104 binding, rather than a species-specific state of the loader alone. Thus, in addition to sterically blocking partner binding, 77ORF104 remodels a critical self-assembly element in DnaI to further interfere with loader self-association.

Given that the N-terminal domain of DnaC/I-type helicase loaders contains the portion of the protein known to bind the replicative helicase, we reasoned that the 77ORF104 inhibitor might not disrupt the ability of DnaI to associate with its cognate target, DnaC (known as *Sa*DnaC, a DnaB-family helicase not to be confused with the *E. coli* DnaI homolog and helicase loader, *Ec*DnaC). To test this idea, we performed pull-down experiments using tagged 77ORF104 as bait to bind either untagged *Sa*DnaI or *Sa*DnaC-bound *Sa*DnaI as prey (Figure 4C). Analysis of the reactions by SDS-PAGE revealed that both free *Sa*DnaI and 77ORF104-bound *Sa*DnaI were able to bind to the *Sa*DnaC helicase equally well. By contrast, 77ORF104 did not bind to the *Sa*DnaC helicase alone. Overall, this result is consistent with a model in which 77ORF104 inhibits *Sa*DnaI function not by blocking loader/helicase associations, but by preventing loader self-assembly. Both light-scattering and analytical size-exclusion chromotography data corrobate this model (Figure 4—figure supplement 1; Figure 4—figure supplement 2).

### 77ORF104 inhibits ssDNA-stimulated ATP hydrolysis by SaDnaI

**Figure 5.**
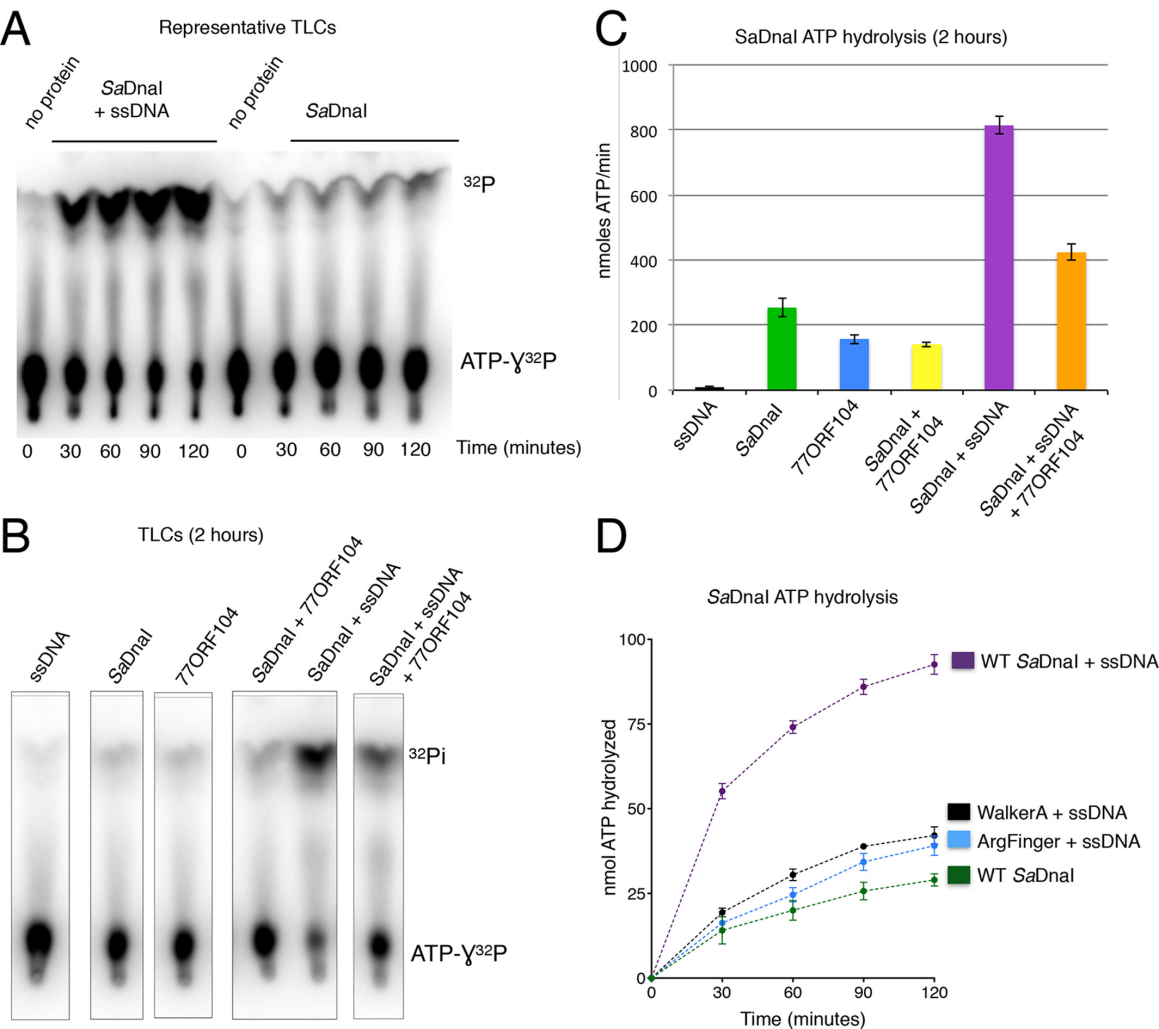
77ORF104 inhibits ssDNA stimulation of ATP hydrolysis by *Sa*DnaI. (**A**) Representative TLC image shown for *Sa*DnaI ATP hydrolysis in the presence (left) or absence (right) of M13ssDNA. (**B**) M13ssDNA stimulates ATP hydrolysis by *Sa*DnaI while 77ORF104 inhibits this effect. (**C**) Representative TLC image of *Sa*DnaI ATP hydrolysis experiments in the presence and absence of wild-type 77ORF104 and/or M13ssDNA. (**D**) Effects of ATPase mutations on observed hydrolysis activity. Walker-A (K170A) and arginine finger (R288A) mutations in *Sa*DnaI show reduced stimulation of activity, comparable to that seen in the absence of ssDNA. **DOI:** http://dx.doi.org/10.7554/eLife.14158.010

If 77ORF104 targets the ability of *Sa*DnaI to self-associate, as opposed to an interaction with the host helicase, then the protein should be expected to affect functions of DnaI that rely on loader-loader interactions. The nucleotide binding cycle of bacterial helicase loaders has been proposed to be coupled to an ability of the loader to self-assemble (Biswas et al., 2004; Mott et al., 2008; Tsai et al., 2009a), an activity that can be stimulated in DnaI/DnaC-family loaders by single-stranded (ss) DNA (Davey et al., 2002; Ioannou et al., 2006). To test whether 77ORF104 might impact such a function, we carried out radioactive ATP hydrolysis assays using [γ-^32^P]-ATP and M13-ssDNA. Although *Sa*DnaI was found to exhibit relatively modest ATPase activity on its own, the presence of M13-ssDNA markedly stimulated nucleotide turnover (Figure 5A). By contrast, when incubated with the 77ORF104 inhibitor in the presence of M13-ssDNA, the observed stimulation of ATP hydrolysis by ssDNA was much reduced (Figures 5B,C). To establish that the increase in ATPase activity we observed was derived from *Sa*DnaI and not from a potentially contaminating ATPase, we cloned, expressed and purified several active site mutants of *Sa*DnaI. Both a Walker A mutant (K170A) and an arginine finger mutant (R288A) showed reduced ATP hydrolysis activity in the presence of ssDNA, indicating that the ssDNA-stimulated activity seen in our assays was indeed specific to DnaI and did not arise from a contaminating ATPase (Figure 5D). Taken together with our structural and biochemical data (see also Figure 4—figure supplement 1), our findings support a model in which 77ORF104 inhibits *Sa*DnaI activity by blocking loader self-assembly and preventing proper ATPase function.

### Evidence for a phage-encoded hijacking mechanism that targets the host helicase

**Figure 6.**
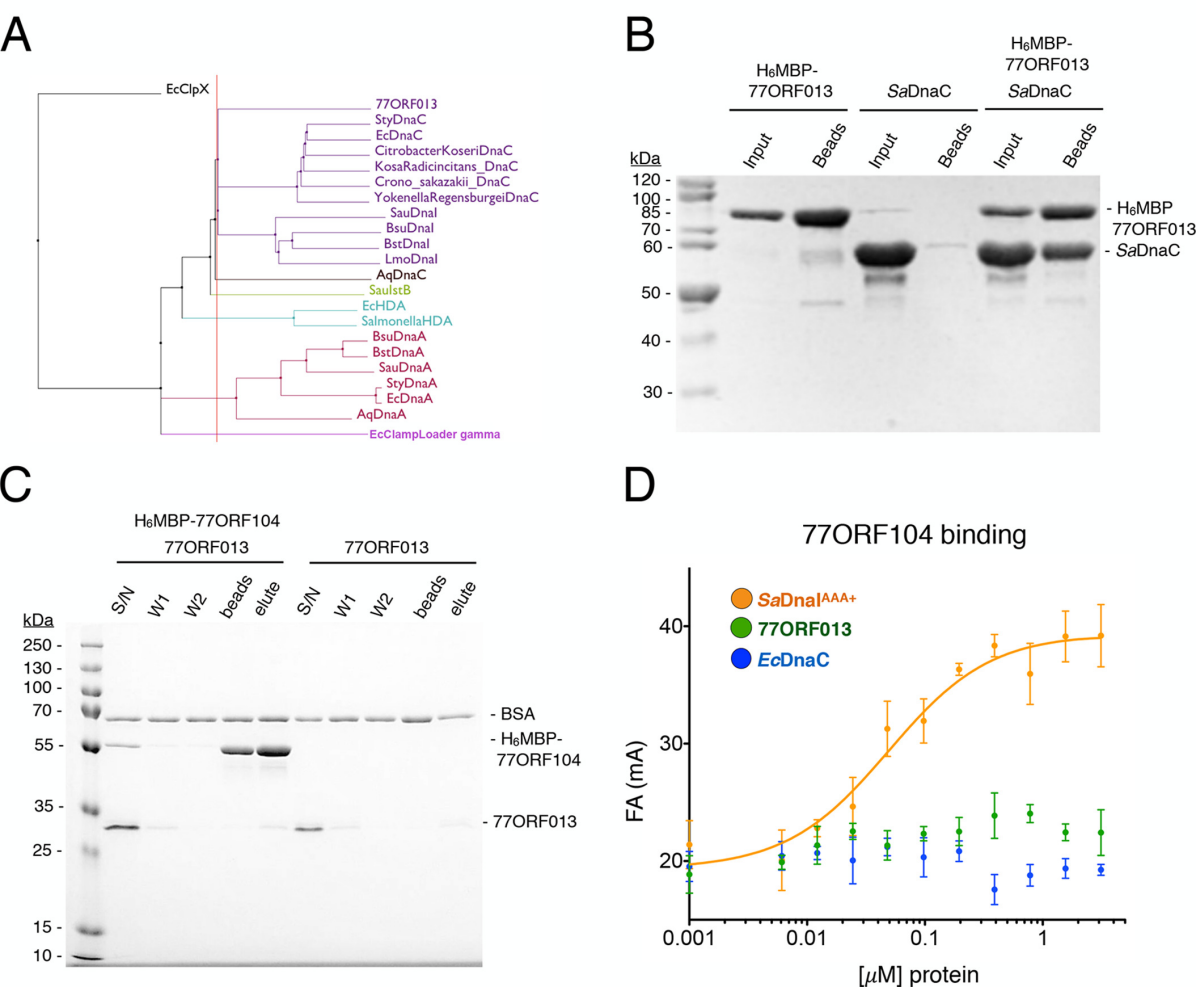
Phage 77 encodes a bacterial helicase loader homolog (ORF013) that binds to the host *Sa*DnaC replicative helicase. (**A**) Phylogenetic tree of the phage encoded *ORF013* gene with various initiator/loader clade AAA+ ATPases. 77ORF013 clusters with DnaC/DnaI family helicase loaders; a distantly related AAA+ ATPase (*E. coli* ClpX) was included for the purpose of rooting the tree. (**B**) SDS-PAGE analysis of amylose pull-down experiments using His_6_MBP-tagged phage loader 77ORF013 and the *S. aureus* DnaC replicative helicase. The 77ORF013 helicase loader homolog binds to the host *Sa*DnaC helicase. (**C**) SDS-PAGE analysis of amylose pull-downs using His_6_MBP-tagged 77ORF104 and 77ORF013 helicase loader homolog. 77ORF013 does not associate with the 77ORF104 inhibitor protein. (**D**) Binding to phage 77ORF104 inhibitor protein to *Sa*DnaI^AAA+^, 77ORF013 and *E. coli* DnaC as measured by a change in fluorescence anisotropy (ΔFA – change in milli-anisotropy units). The X-axis represents protein concentration. Data points and error bars derive from three-independent experiments. No measurable binding was observed for the phage 77 ORF013 or *E. coli* DnaC. *Sa*DnaI^AAA+^ is shown as a positive control. **DOI:** http://dx.doi.org/10.7554/eLife.14158.011

Because phage 77 utilizes a specialized protein to inhibit the activity of the host’s helicase loader, we became curious as to how the phage itself might replicate. Interestingly, within the phage 77 genome, the gene next to *ORF104* (termed *ORF013*) is annotated as an AAA+ ATPase. The two genes are located in the same operon in the phage chromosome, suggesting that they might be expressed contemporaneously and function synergistically. To determine the protein family to which 77ORF013 belongs, we searched the database for homologous proteins of known function. Surprisingly, this analysis revealed that the *77ORF013* gene encodes a putative DnaC/DnaI-type helicase loader (Figure 6A), thereby raising the possibility that the 77ORF013 protein might bind to the host’s replicative helicase directly. To test this hypothesis, we cloned, expressed and purified a tagged version of 77ORF013 and performed amylose pull-down experiments using the untagged *Sa*DnaC replicative helicase as prey. SDS-PAGE analysis of different pull-down fractions showed that the phage 77 ORF013 protein was indeed capable of binding the *Sa*DnaC replicative helicase (Figure 6B); analytical gel filtration chromatography studies corroborate this result (Figure 4—figure supplement 2). Thus, the partner open reading frame shared by the 77ORF104 inhibitor protein appears to encode for a phage variant of a bacterial replicative helicase loader.

The finding that 77ORF013 is a helicase loader homolog raised the question as to whether the cognate 77ORF104 protein would bind to it as well as to the host *Sa*DnaI protein. To address this question, we performed pull-downs of the purified tagged 77ORF104 inhibitor with 77ORF013. This assay revealed no association between the putative phage-encoded helicase loader and 77ORF104 (Figure 6C). As measured by anisotropy, 77ORF104 also proved unable to interact with either a Gram-negative, DnaC-type helicase loader (*E. coli* DnaC) or 77ORF013 (Figure 6D), indicating that the association of 77ORF104 with DnaI is specific to the *S. aureus* helicase loader. Together, these findings indicate that the phage inhibitor protein’s association with *Sa*DnaI is specific for binding only to the helicase loader of a host bacterium targeted by the virus.

## Discussion

In the present study, we have set out to better understand not only the function of DnaI-family bacterial helicase loaders, but also how bacteriophage can interfere with helicase loader activity to block host DNA replication. We determined crystal structures of the AAA+ ATPase region of the *Sa*DnaI helicase loader in a nucleotide-free state and bound to both the ATP mimic ADP•BeF_3_ and a DnaI-binding protein (77ORF104) from phage 77 (Figures 3 and 4A–B). The structures reveal that binding of the phage inhibitor not only remodels a region known to be critical for DnaI homo-oligomerization, the Initiator Specific Motif (ISM) (Figure 4B), but that it also sterically occludes a principal subunit-subunit interaction surface on the helicase loader (Figure 4A). Association studies between 77ORF104 and *Sa*DnaI show that productive interactions are not dependent on nucleotide binding (Figure 2C), but instead require the last five C-terminal residues of the phage inhibitor protein and a particular tyrosine residue (Tyr17) on the inhibitor protein that is buried in the binding interface with *Sa*DnaI (Figure 3C–D).

The observed structural changes and regions masked by 77ORF104 binding would be expected to prevent DnaI self-association. This prediction is borne out by ATPase assays showing that this activity – which relies on DnaI-DnaI interactions to form a competent catalytic center – is repressed by the protein (Figure 5B). Interestingly, ORF104 was not found to disrupt the loader’s association with the replicative helicase *Sa*DnaC (Figure 4C). This result indicates that 77ORF104 could in principle act at multiple stages during the DNA replication cycle where helicase-loading events are utilized, including both initiation and replication restart (Bell and Kaguni, 2013; Heller and Marians, 2006).

During the course of carrying out this work, we discovered that phage 77 encodes another protein, ORF013, which is homologous to other DnaC/I-family members (Figure 6A–B). Binding studies revealed that this protein binds to the host’s replicative helicase, *Sa*DnaC, but not to the inhibitory 77ORF104 protein that is also produced by phage 77 (Figure 6C–D). These observations suggest that the 77ORF013 and 77ORF104, which appear to both share a common operon, may serve as a two-pronged mechanism by which host replication can be inhibited and a portion of the cell’s replication machinery co-opted for promoting viral DNA duplication. Surveys of other *S. aureus* phage genomes, such as phages 80 and 80α, reveal the existence of homologs of both the phage 77 ORF013 protein and the ORF104 inhibitor (Kwan et al., 2005; Liu et al., 2004; Tormo-Más et al., 2010), indicating that the phage 77 ORF013/ORF104 system may be widespread among *Staphylococcus aureus* phages. The hijacking of a host replicative helicase for viral replication has been seen before with divergent viruses, including the phages λ, P2 and Mu, as well as influenza (Kawaguchi and Nagata, 2007; Kruklitis and Nakai, 1994; Mallory et al., 1990; Odegrip et al., 2000). However, the co-expression of a putative viral helicase loader with an inhibitor of a host helicase loader has only been seen thus far for phage 77 and its relatives. Although replication initiation has yet to be reconstituted for *S. aureus* using purified factors, the planned development of such a system will allow for future investigations into the effects of ORF013/ORF104 family proteins on helicase loading and phage replication in vitro.

**Figure 7.**
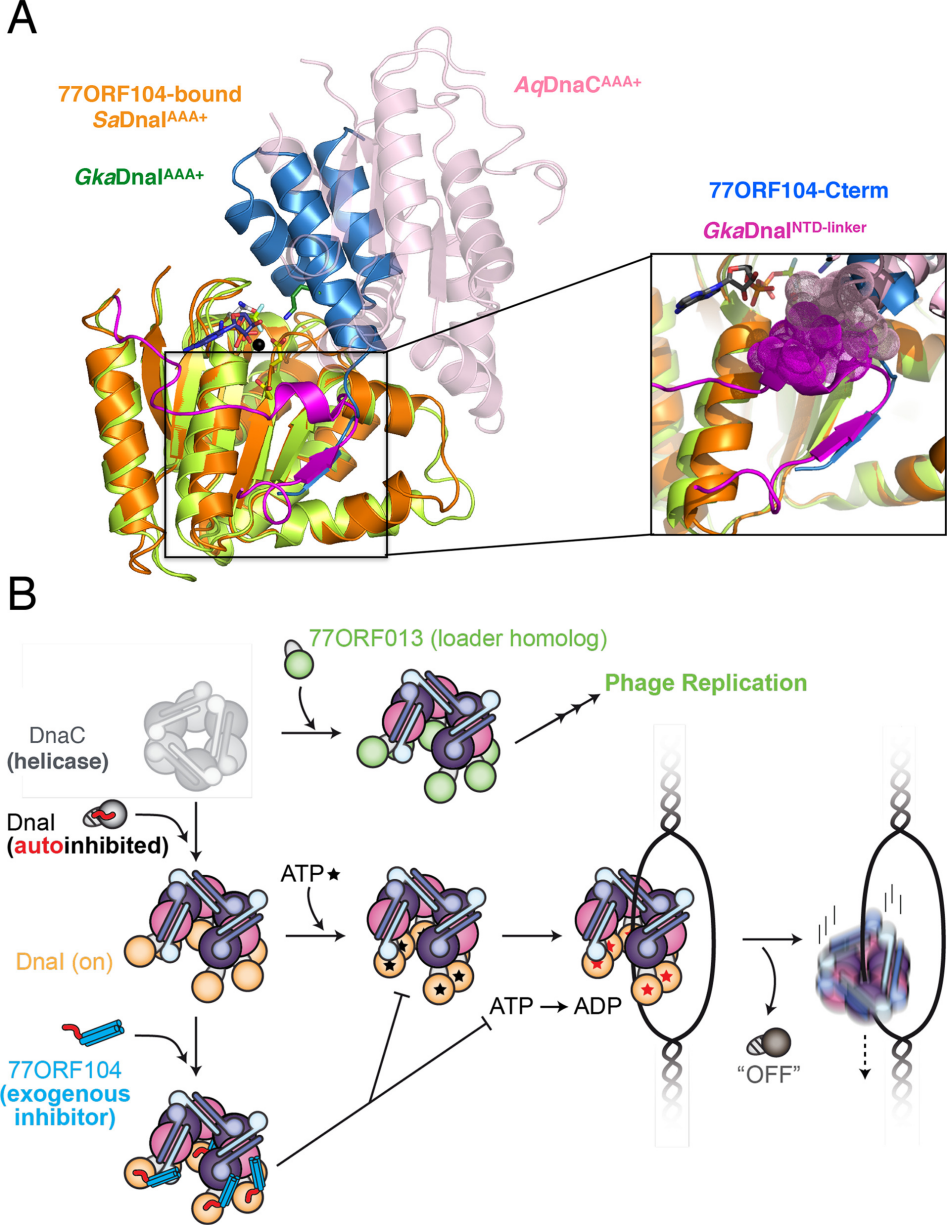
Insights into bacterial helicase loader mechanism and regulation derived from this study. (**A**) The N- and C-terminal domain linker of DnaC/I helicase loaders occupies a region bound by the phage 77 inhibitor protein. The monomeric ATPase domain of *G. kaustophilus* DnaI (*Gka*DnaI ^AAA+^) (PDB ID 2W58, [Tsai et al., 2009b]) is shown superposed onto a nucleotide-assembled dimer of both the *A. aeolicus* DnaC AAA+ domain (a construct that lacks the linker, PDB ID 3ECC, [Mott et al., 2008])) and the *Sa*DnaI^AAA+^ domain (as seen in complex with 77ORF104). The C-terminal tail of 77ORF104 forms a β-strand that occupies the same location as the *Gka*DnaI^AAA+^ linker (magenta), although the strand runs in the opposite direction. (Inset) Close-up view of the linker/tail binding site. Several amino acids in the linker are shown as space filling (magenta) to illustrate how this element would sterically clash with a second, incoming helicase loader ATPase domain to prevent self assembly (pink spheres, from *Aq*DnaC). (**B**) Schematic summarizing the auto-regulation of DnaC/I helicase loaders and the ability of phage 77-family viruses to inhibit host helicase loading and co-opt host replicative helicases in *S. aureus* and related Gram-positive bacteria. The bacterial helicase loader linker region forms an intra-molecular interaction with its associated AAA+ domain to auto-repress inopportune loader/loader interactions; upon binding to the replicative helicase, the linker is proposed to undock from this region to allow self-assembly of the loader ATPase folds and subsequently assist with loading of the replicative helicase onto single-stranded origin DNA. The phage 77 ORF104 inhibitor protein binds to cognate loaders such as *Sa*DnaI, repressing loader self-association, ATPase activity, and presumably its ability to properly participate in the helicase loading reaction. Phage 77 is also found to encode a helicase loader homolog (*ORF013*) that directly binds to the host replicative helicase, likely as a means to re-direct the host’s replication machinery toward replication of the viral genome. **DOI:** http://dx.doi.org/10.7554/eLife.14158.012

A second unexpected finding obtained from this work derives from an analysis of where 77ORF104 binds to *Sa*DnaI in the context of other bacterial helicase loading proteins. Previous studies of *Bacillus subtilis* DnaI and the related *E. coli* DnaC protein have shown that bacterial helicase loaders typically require helicase binding and/or ssDNA binding to promote ATPase activity (Ioannou et al., 2006; Learn et al., 1997; Davey et al., 2002), a function proposed to directly depend on loader self assembly (Mott et al., 2008). Superposition of the 77ORF104-*Sa*DnaI complex with structures of other DnaI orthologs, particularly those that contain a portion of the helicase loaders’ linker regions, reveal that the C-terminal tail of the inhibitor actually occupies a pocket that corresponds to the observed binding site for a portion of the linker itself (Figure 7A). Moreover, inspection of these overlays shows that in associating with its own AAA+ ATPase domain, the inter-domain linker additionally blocks a portion of the surface where subunit-subunit interactions form during loader self-association (Figure 7A, see inset). This observation explains why DnaI ATPase domain constructs containing a portion of the linker crystallized as monomers even in the presence of nucleotide, and indicates that the linker likely serves as a switch that reports on relative status of the N- and C-terminal regions, repressing loader-loader interactions until the correct helicase and DNA targets are bound (Figure 7B). In this regard, the 77ORF104 protein exploits this binding locus, taking advantage of the linker-binding pocket to inactivate the host loader, an activity that would allow the cellular replicative helicase to be re-routed by the phage-encoded loader to promote viral replication (Figure 7B). This interaction, which occurs within a small hydrophobic pocket on the loader AAA+ ATPase domain, suggests that the linker-binding region of the DnaI AAA+ domain could serve as an attractive site for the development of chemical inhibitors to target helicase loading and DNA replication in bacteria. Future efforts will help test and establish these concepts further.

## Materials and methods

### Cloning, expression and protein purification

Coding sequences for full-length *Staphylococcus aureus* DnaI, DnaI^AAA+^ (residues 136–306), DnaI^NTD^ (residues 1–117), *Sa*DnaC replicative helicase, and phage 77 ORF104 and ORF013 were cloned into a pET28b (EMD Millipore, Billerica, MA) derivative with a tobacco etch virus (TEV) protease‐cleavable, N‐terminal hexahistidine-MBP tag and verified by sequencing (Elim Biopharmaceuticals, Hayward, CA). All *Sa*DnaI and phage 77 proteins were expressed in *E. coli*, using BL21 codon-plus (DE3) RIL cells (Agilent), by inducing at A_600_= 0.4–0.5 with 1 mM IPTG at 37°C for 3 hr. Cells were harvested by centrifugation, resuspended in lysis buffer and lysed by sonication. All *S. aureus* DnaI and phage proteins were purified by Ni^2+^‐affinity chromatography over a 5‐mL HisTrap HP column (GE). Lysis buffer consisted of 500 mM NaCl, 20 mM imidazole, 50 mM HEPES-KOH pH 7.5, 10 mM MgCl_2_, 10% glycerol, and protease inhibitors (1 mM phenylmethylsulfonyl fluoride (PMSF), 1 mg/mL Pepstatin A, 1 mg/mL Leupeptin). After washing in binding buffer containing 1 M NaCl, proteins were eluted with 300 mM imidazole in elution buffer containing 50 mM NaCl, 50 mM HEPES-KOH pH 7.5, 10 mM MgCl_2_, 10% glycerol and protease inhibitors (1 mM PMSF, 1 mg/mL Pepstatin A, and 1 mg/mL Leupeptin) over 5 column volumes. His_6_-MBP tags were removed with His_6_-tagged TEV protease (MacroLab, UC Berkeley) adding the TEV protease at a ratio of 1:40 TEV:protein and incubating at 4°C overnight. Proteins were then exchanged into binding buffer, repassaged over a HisTrap column, and run over a Sepharose S200 gel filtration column (GE) in 500 mM NaCl, 50 mM HEPES-KOH pH 7.5, 10 mM MgCl_2_, 10% glycerol, and protease inhibitors (1 mM PMSF, 1 mg/mL Pepstatin A, and 1 mg/mL Leupeptin). Selenomethionine labeled proteins were purified by the same protocol with the exception that 0.5 mM TCEP was added to all buffers. Protein purity was assessed by polyacrylamide gel electrophoresis and Coomassie staining. Proteins were concentrated by centrifugal ultrafiltration (EMD Millipore Amicon Ultra). Concentration was determined by absorption at 280 nm using the following calculated extinction coefficients: 23,380 M^-1^ cm^-1^ for FL *Sa*DnaI, 72,310 M^-1^ cm^-1^ for H_6_MBP-77ORF104, 14,440 M^-1^ cm^-1^ for *Sa*DnaI^AAA+(136–306)^, 4470 M^-1^ cm^-1^ for 77ORF104 and 7450 M^-1^ cm^-1^ for *Sa*DnaI^NTD(1–117)^, 15,930 M^-1^ cm^-1^ for *Sa*DnaI^CTD(117–306)^, 28,880 M^-1^ cm^-1^ for 77ORF013, and 27,850 M^-1^ cm^-1^ for *Sa*DnaC replicative helicase. Mutant *Staphylococcus* bacteriophage 77ORF104 and *S. aureus* DnaI proteins were generated using QuickChange (Agilent) site‐specific mutagenesis and sequenced (GeneWiz, Frederick, MD). *S. aureus* DnaI and phage 77ORF104 mutants were purified as described above.

### Expression and purification of the *S. aureus* replicative helicase, SaDnaC

His_6_-MBP tagged *Sa*DnaC was expressed in *E. coli*, using BL21 codon-plus (DE3) RIL cells (Agilent), by induction at A_600 _= 0.6 with 1 mM IPTG at 37°C for 2.5–3 hr. Cells were harvested by centrifugation, resuspended in lysis buffer and lysed by sonication. *Sa*DnaC was purified by Ni^2+^‐affinity chromatography over a 5‐mL HisTrap HP column (GE). Lysis buffer consisted of 500 mM NaCl, 20 mM imidazole, 50 mM Tris-HCl pH 8.0, 2 mM MgCl_2_, 10% glycerol, 0.5 mM β-mercaptoethanol and protease inhibitors (1 mM PMSF, 1 mg/mL Pepstatin A, 1 mg/mL Leupeptin). After washing in binding buffer containing 500 mM NaCl, proteins were eluted with 300 mM imidazole in elution buffer containing 50 mM NaCl, 50 mM Tris-HCl pH 8.0 2 mM MgCl_2_, 10% glycerol, 0.5 mM β-mercaptoethanol and protease inhibitors over 5 column volumes. The elution was then loaded onto a HiTrap Mono Q Sepharose HP Column (GE Healthcare, United Kingdom) equilibrated in 150 mM NaCl, 50 mM Tris-HCl pH 8.0, 2 mM MgCl_2_, 10% glycerol, 0.5 mM β-mercaptoethanol and protease inhibitors and eluted in buffer containing 300 mM NaCl. The His_6_-MBP tag was removed with His_6_-tagged TEV protease adding the TEV protease at a ratio of 1:40 TEV:protein and incubating at 4°C overnight. Proteins were then exchanged into binding buffer, repassaged over a HisTrap column, and run over a Sepharose S300 gel filtration column (GE) in 800 mM NaCl, 20 mM Tris-HCl pH 8.0, 5 mM MgCl_2_, 10% glycerol, 1 mM β-mercaptoethanol, 0.01 mM ATP and protease inhibitors. Protein purity was assessed by polyacrylamide gel electrophoresis and Coomassie staining. Proteins were concentrated by centrifugation (Millipore Amicon Ultra MWCO 30 K). Concentration was determined by absorption at 280 nm using a calculated extinction coefficient of 27,850 M^-1^ cm^-1^ for the *Sa*DnaC replicative helicase.

### Crystallization of an ADP•BeF_3_-bound SaDnaI^AAA+^•77ORF104 complex

To crystallize the ADP•BeF_3_-bound *Sa*DnaI^AAA+^*•*77ORF104 complex, we expressed and purified both proteins individually, using minimal media containing selenomethionine for *Sa*DnaI^AAA+^ and 2xYT media for 77ORF104. Following purification, the *Sa*DnaI^AAA+^*•*77ORF104 complex was formed using a two-fold excess of 77ORF104 to *Sa*DnaI^AAA+^, and the complex purified by passage over a Sepharose S200 gel filtration column equilibrated in S200 buffer plus 0.5 mM TCEP. Following concentration to 6 mg/mL, ADP•BeF_3_was spiked into the sample to 2 mM final concentration as an ATP mimetic. Crystallization was performed by hanging-drop vapor diffusion by mixing 2 μL of protein complex in S200 sizing buffer with 2 μL well solution (50 mM Tris-HCl 8.5, 20 mM MgCl_2_, 20% ethanol). Large, rod-shaped crystals grew within 1–2 days at 21°C using a protein concentration of 6.5 mg/mL. Crystals were cryo-protected by serial exchange into a harvesting buffer containing 25% PEG 400, 50 mM HEPES 7.5, 500mM NaCl, 5 mM MgCl_2_, 10% glycerol, 0.5 mM TCEP, and 2 mM ADP•BeF_3_ before flash freezing in liquid nitrogen. Crystals were stored in liquid nitrogen prior to data collection.

### Crystallization of apo SaDnaI^AAA+^

Apo *Sa*DnaI^AAA+^ was expressed and purified as described above with only a minor change made to the S200 sizing buffer (substituting 500 mM KCl for 500 mM NaCl). Sparse matrix screening was performed as described above at a protein concentration of 5 mg/mL. The final crystallization conditions for *Sa*DnaI^AAA+^ contained a well solution of 0.1 M citric acid, 10 mM MgCl_2_, and 0.8 M ammonium sulfate at pH 4.0. Prior to crystallization, ADP•BeF_3_ was spiked into the sample to 2 mM final concentration and crystals were grown at 21°C. Crystals were cryo-protected by serial exchange into a harvesting buffer containing 25% glycerol, 0.1 M citric acid pH 4.0, 0.8 M ammonium sulfate, 50 mM HEPES-KOH pH 7.5, 10 mM MgCl_2_, 10% glycerol, and 2 mM ADP•BeF_3_ before flash freezing in liquid nitrogen.

### Data collection for ADP•BeF_3_-SaDnaI^AAA+^•77ORF104 and apo SaDnaI^AAA+^

Datasets for both structures were collected at beamline X25 at the National Synchrotron Light Source (NSLS), Brookhaven National Laboratory. A single selenomethionine SAD dataset for the *Sa*DnaI^AAA+^*•*77ORF104 complex was collected at a wavelength of 0.979 Å and processed in XDS (Kabsch, 1988, 2010) (Table 1). One ADP•BeF_3_*-Sa*DnaI^AAA+^*•*77ORF104 complex was found to occupy each asymmetric unit. Selenium positions for the SeMet-*Sa*DnaI^AAA+^were determined using HYSS (Hybrid Substructure Search) as part of the PHENIX package (Adams et al., 2010; Grosse-Kunstleve and Adams, 2003). Initial experimental electron density maps were generated from AUTOSOL (Terwilliger et al., 2009) via phasing by single wavelength anomalous dispersion. Several cycles of model building were performed using COOT (Emsley and Cowtan, 2004) and the PHENIX package was used for refinement (Adams et al., 2010).

The apo *Sa*DnaI^AAA+^ dataset was collected at a wavelength of 1.5 Å and processed in XDS (Kabsch, 1988, 2010). Crystals were determined to belong to the space group P2_1_2_1_2_1_ with unit cell dimensions a=113.09 Å, b=126.26 Å, c=183.34 Å, and a solvent content of approximately 52.4% (Table 1). Twelve *Sa*DnaI^AAA+^ molecules were found in the asymmetric unit. The structure was solved by molecular replacement using MR-PHASER in the PHENIX package (McCoy et al., 2007) followed by several cycles of model building in COOT (Emsley and Cowtan, 2004) and refinement in PHENIX (Adams et al., 2010). The minimal AAA+ core domain of *Sa*DnaI from the complex structure, lacking the ISM, was used as a search model with MR-PHASER. The final, 12-chain model converged at an R_work_/R_free_ of 22.5/26.9% for the resolution range of 48.1–2.6Å (Adams et al., 2010). Each chain contains residues 136–306 *Sa*DnaI^AAA+^ and a single SO_4_^2-^ ion modeled into the P-loop of the AAA+ ATPase domain. Despite the inclusion of Mg^2+^ ions and ADP•BeF_3_ with the protein prior to crystallization, no density for either moiety could be observed, likely because the low pH and high SO_4_ ion concentration present in the well solution precluded stable binding. The coordinates for the ADP•BeF_3_*-Sa*DnaI^AAA+^*•*77ORF104 complex (5HE9) and apo *Sa*DnaI^AAA+^ (5HE8) structures have been uploaded to the PDB.

### Limited trypsin proteolysis of *S. aureus* DnaI in the presence of 77ORF104

Both *Sa*DnaI and 77ORF104 proteins were dialyzed overnight into a reaction buffer containing 500 mM KCl, 50 mM HEPES-KOH pH 7.5, 10 mM MgCl_2_ and 10% glycerol. *Sa*DnaI alone or *Sa*DnaI in the presence of the 77ORF104 protein were incubated on ice for 10 min prior to addition of trypsin. Trypsin protease was added to the reaction resulting in the final concentrations: 5 μM, 10 μM, 20 μM and 80 μM; proteins were then incubated with trypsin for 30 min at 25°C. Reactions were quenched with 200 μM PMSF. After addition of an equal volume of 2X SDS-loading dye, reactions were heated at 95°C for 5 min before running 15 μL of each reaction on a 15% SDS-PAGE gel and staining with Coomassie blue.

### Amylose pull-downs

Amylose pull-downs were performed using His_6_MBP-tagged proteins and untagged proteins in a total volume of 200 μL. All His_6_MBP-tagged proteins (164 µM) were dialyzed into binding buffer containing 20 mM HEPES-KOH pH 7.5, 100 mM NaCl, 5 mM MgCl_2_, 10% glycerol, and 1 mM DTT at 4°C prior to setting up the protein binding reaction. Pulldowns that included ATP had a final ATP concentration of 2 mM. H_6_MBP-tagged proteins were incubated with their untagged protein for 10 min at 25°C before the addition of 50 μL of amylose bead slurry, pre-equilibrated in binding buffer. The final reactions contained 10 µM His_6_MBP-tagged protein and 20 µM untagged protein. After the addition of beads, the binding reaction was incubated for 20 min at 25°C, with gentle mixing every 2 min during the incubation. Amylose binding reactions were spun down at 1500 RPM for 1 min. Amylose beads were then washed twice prior to either elution with maltose elution buffer (40 mM maltose, 20 mM HEPES-KOH pH 7.5, 100 mM NaCl, 5 mM MgCl_2_, 10% glycerol, and 1 mM DTT) or the addition of 2X SDS loading dye. Amylose pull-down supernatant, washes, elution and beads samples were run on a 12% SDS-PAGE gel (a slight excess of total protein was used over the binding capacity of the bead volume, so as to allow for visualization of sample input). SDS-PAGE gels were stained for 30 min with SYPRO Orange stain diluted to 1X in 10% glacial acetic acid. Gels were washed with dI-H_2_0 three times before imaging the gels on a Kodak Gel Imager.

### N-terminal labeling of 77ORF104

Purified wild-type 77ORF104 protein was exchanged into amine-labeling buffer (50 mM HEPES-KOH pH 7.5, 500 mM KCl, 10% glycerol, 10 mM MgCl_2_, 1 mM β-mercaptoethanol) by centrifugation. The neutral pH favors labeling of the amino terminus of proteins rather than surface lysines (Sélo et al., 1996). The protein was then concentrated to a final volume of 500 μL by centrifugation using 500μL VIVASPIN ultrafiltration units (MWCO 3K, Sartorius) to a final concentration of 342 μM. AlexaFluor 488 5-SDP (sulfodicholorphenol) ester (Life Technologies) (1 mg) was dissolved into 20 μL DMSO. The dye (20 µL) was added to the concentrated protein (342 μM) and the reaction incubated at 4°C while wrapped in aluminum foil and rocking for 1 hr. Unreacted dye was quenched by adding 20 μL of 1 M L-lysine in 20 mM Tris-HCl pH 7.5. Reactions were incubated with the quench for 30 min at 25°C. Free dye and quench were separated from dye-protein conjugates using a 10 mL PD-10 desalting column (GE) equilibrated in the amine-labeling buffer (see above). The labeled protein was then exchanged into a buffer containing 50 mM HEPES-KOH pH 7.5, 500 mM KCl, 30% glycerol, 10 mM MgCl_2_, 1 mM β-mercaptoethanol by centrifugation. The concentrated labeled 77ORF104 protein was aliquoted, snap frozen in liquid nitrogen, and stored at -80°C.

### Anisotropy-based competition assays and protein-protein binding assays

Purified proteins were prepared in 2-fold dilutions steps in dilution buffer containing 50 mM HEPES-KOH pH 7.5, 500 mM KCl, 10% glycerol and 10 mM MgCl_2_. N-terminally labeled, wild-type 77ORF104 was diluted to 40 nM in a reaction buffer (50 mM HEPES-KOH pH 7.5, 10% glycerol, 5 mM MgCl_2_, 1 mM DTT) to make a 2X stock. For serial dilutions, purified proteins were sequentially diluted in 2-fold steps into protein dilution buffer. Proteins were mixed with 10 µL of N-terminally labeled 77ORF104 on ice and incubated for 10 min. The final reaction volume was 20 μL containing the final buffer conditions (50 mM HEPES-KOH pH 7.5, 125 mM KCl, 10% glycerol, 5 mM MgCl_2_, 1 mM DTT). The final concentration of labeled 77ORF104 in each reaction was 20 nM. Anisotropy measurements were recorded using CLARIOStar microplate reader (BMG LAB TECH) at 535 nm. All data points are the average of three independent measurements. For the 77ORF104 protein binding assays, data were plotted using GraphPad Prism Version 6 (GraphPad Prism Software, La Jolla California USA, www.graphpad.com) and fit by nonlinear regression to the single-site binding equation (Swillens, 1995).

For the 77ORF104 competition experiments, reactions were prepared as previously described except that the reaction mixture contained 20 nM labeled 77ORF104 and 2 μM *Sa*DnaI^AAA+^. Wild-type 77ORF104 and protein mutants were serially diluted (as described above) in the same dilution buffer and final buffer solution. Assays were performed as described above for the 77ORF104 mutants. Anisotropy measurements were recorded using a CLARIOStar microplate reader (BMG LAB TECH). All data points represent the average of three independent measurements. Error bars represent the standard deviation of three independent measurements. Data points were plotted using GraphPad Prism Version 6 and fit with a simple smooth line for aiding visualization.

### Radioactive ATP hydrolysis assays

ATPase assays were performed in 30 µL of reaction buffer containing: 10 nM (4500 Ci/mmol) [**γ**^32^P]ATP, 100μM cold ATP, 100 mM KCl, 50 mM HEPES-KOH pH 7.5, 10 mM MgCl_2_, 10% glycerol, and M13mp18 ssDNA (New England Biolabs, Inc.) added to a final concentration of 80 ng/uL. The final reactions contained 10 µM *Sa*DnaI and 60 µM 77ORF104. The 77ORF104 or *Sa*DnaI mutant proteins were added to the reactions on ice, after which the tubes were shifted to 37°C for 2 hr. 3 µL was next removed from the reaction and quenched by the addition of 3 µL of 250 mM EDTA pH 8.0 and 1% SDS. Quenched reactions were spotted (1 µL) onto thin-layer chromatography sheets coated with polyethyleneimine cellulose (PEI-Cellulose F; EM Science) and developed in 0.4 M potassium phosphate (pH 3.4) for 30 min. [**γ**^32^P] ATP and free phosphate [**γ**^32^P] migrated differently and were quantitated using a Typhoon FLA 9500 PhosphorImager (GE) and ImageJ software (Schneider et al., 2012).

### Supplemental information

#### Centrifugal and dynamic light scattering assays for phage 77 ORF104 disruption of SaDnaI oligomerization

To further test whether the 77ORF104 inhibitor directly disrupts the ability of *Sa*DnaI to self-oligomerize into filaments, we looked at loader interactions using a simple *Sa*DnaI pelleting assay (Figure 4—figure supplement 1A). When chilled on ice at elevated concentrations, *Sa*DnaI oligomerizes into visible microcrystals that can be sedimented after a brief centrifugation step. If the inhibitor were to directly disrupt DnaI-DnaI contacts, we reasoned that it should keep the loader in solution. *Sa*DnaI pelleting assays were performed in 60 µL of reaction buffer containing 20 mM HEPES-KOH pH 7.5, 100 mM NaCl, 10% glycerol, 5 mM MgCl_2_g and 6 mM ATP. The final reactions contained 90 µM *Sa*DnaI and 180 µM 77ORF104. Reactions were incubated for 20 min on ice before being spun down at 14,000 x g for 1 min. The starting material, pellet and supernatant were then assessed by SDS-PAGE. In line with our predictions, the loader remains in solution when incubated with the inhibitor (Figure 4—figure supplement 1A).

To better look at the propensity of DnaI to self-associate and of the ORF104 protein to affect this behavior, we next turned to dynamic light scattering (DLS, model DynaPro NanoStar from Wyatt Technology), which can report on the approximate radii of gyration, relative masses, and polydispersity of macromolecules in solution. Dynamic light scattering (DLS) experiments were performed at 10˚C in 70 µL reaction buffer containing 50 mM HEPES-KOH pH 7.5, 500 mM NaCl, 10% glycerol, 5 mM MgCl_2_, 1 mM DTT, and 2 mM ATP. DLS reactions were prepared at a stoichiometric ratio of 1:1 *Sa*DnaI to 77ORF104 with final concentrations of 90 µM SaDnaI and 90 µM 77ORF104. The resultant data show that on its own, *Sa*DnaI forms a very broad, polydisperse mixture of species with a large average molecular weight (Figure 4—figure supplement 1B,C). By contrast, when measured in the presence of the inhibitor protein, a much smaller, less polydisperse species is formed. Interestingly, the average molecular weight calculated for the dominant species in the DnaI-ORF104 mixture is larger than expected for a 1:1 complex, suggesting that complex formation is incomplete under the conditions tested here and/or that the N-terminal domain of DnaI might self-associate through unsatisfied, exposed surfaces that would normally be used for binding to the helicase. Nonetheless, these findings corroborate the structural and biochemical data indicating that the phage inhibitor disrupts loader-loader interactions.

#### Analytical sizing of protein complexes

To further probe the interactions between *Sa*DnaC with the phage 77 ORF013 and ORF104 proteins and with *Sa*DnaI, we performed analytical sizing runs on a 5/150 GL Superdex 200 column equilibrated in buffer containing 20 mM Tris-HCl pH 8.0, 200 mM NaCl, 5% glycerol, 5 mM MgCl_2_g, 1 mM BME and 10 mM ATP. Protein samples were prepared at final concentrations of 7 µM SaDnaC helicase, 14 µM SaDnaI loader, 14 µM 77ORF013 and 30 µM 77ORF104. Peak fractions were collected and ran on 12% SDS-PAGE gels in 1X MOPS running buffer. Protein bands were detected by staining with Imperial Protein Stain (ThermoFischer Scientific).

The data from this study (Figure 4—figure supplement 2) show that: **1**) the *Sa*DnaC helicase associates with both the *Sa*DnaI helicase loader and the phage helicase loader homolog 77ORF013 (albeit with a degree of instability, as the bands for the helicase and loader proteins do not perfectly overlap –the peak maxima for ORF013 alone occurs at fraction 44 and for ORF013 plus *Sa*DnaI at fraction 42, and at fractions 42 and 40 for *Sa*DnaI alone and with *Sa*DnaC, respectively), and **2**) that the inhibitor does not affect the migration of either the *Sa*DnaI helicase loader or phage loader 77ORF013 or the *Sa*DnaC helicase. Interestingly, based on standards run on the same column (not shown), the helicase *Sa*DnaC does not appear to form a stable homo-hexameric ring but instead migrates as an apparent dimeric species.

Although tangential to the primary findings of the paper, these results turn out to weigh in on a paradox with respect to how bacterial replicative helicases are loaded. In *B. subtilis*, helicase loading has been reported to occur not by a ring opening mechanism (as is thought to happen in *E. coli)* (Arias-Palomo et al., 2013; Davey and O’Donnell, 2003), but rather by a DnaI-directed assembly process that additionally requires two co-chaperones not found in Gram-negative organisms, DnaB (which is unrelated to the *E. coli* DnaB helicase) and DnaD (Rokop et al., 2004; Smits et al., 2010; Velten et al., 2003). Stable hexamers have been observed for Gram-positive DnaC helicases, but only when using thermophilic proteins (e.g. *B. stearothermophilus* and *G. kaustophilus* [Bailey et al., 2007a; Ioannou et al., 2006; Lo et al., 2009; Soultanas, 2002; Tsai et al., 2009b]), whose room-temperature behavior may differ from their mesophilic counterparts. It should be noted that dimers of DnaB-family helicases have been observed previously, wherein the dimer is stabilized by interactions between the N-terminal 'collar' domains of the protein (Bailey et al., 2007b). Our findings, which show that *S. aureus* helicase/loader interactions form but are somewhat unstable, and that the DnaC helicase does not readily assemble a pre-formed hexamer, are in accord with observations reported for the *B. subtilis* system.

## Additional files

### Major datasets

The following datasets were generated:

Hood IV, Berger JM,2016,Crystal structure of the bacterial helicase loader DnaI,http://www.rcsb.org/pdb/explore/explore.do?structureId=5HE8,Publicly available at the RCSB Protein Data Bank (accession no. 5HE8)

Hood IV, Berger JM,2016,Crystal structure of the bacterial helicase loader DnaI in complex with the 77ORF104 inhibitor and ADP•BeF_3_,http://www.rcsb.org/pdb/explore/explore.do?structureId=5HE9,Publicly available at the RCSB Protein Data Bank (accession no. 5HE9)

The following previously published datasets were used:

Seetharaman J, Chen Y, Wang D, Fang Y, Cunningham K, Ma L-C, Xia R, Liu J, Baran MC, Acton TB, Rost B, Montelione GT, Hunt JF, Tong L, Northeast Structural Genomics ConsortiumCrystal structure of a putative primosome component from Streptococcus pyogenes serotype M3. Northeast Structural Genomics target DR58,http://www.rcsb.org/pdb/explore/explore.do?structureId=2QGZ,Publicly available at the RCSB Protein Data Bank (accession no. 2QGZ)

Tsai KL, Lo YH, Sun YJ, Hsiao CD,2009,Crystal Structure of the DnaI,http://www.rcsb.org/pdb/explore/explore.do?structureId=2W58,Publicly available at the RCSB Protein Data Bank (accession no. 2W58)

Mott ML, Erzberger JP, Coons MM, Berger J,2008,Crystal structure of the DnaC helicase loader in complex with ADP-BeF_3_,http://www.rcsb.org/pdb/explore/explore.do?structureId=3ECC,Publicly available at the RCSB Protein Data Bank (accession no. 3ECC)
