# Supplementary material for: Viral hijacking of a replicative helicase loader and its implications for helicase loading control and phage replication
Source: eLife. 2016 May 31;5:e14158. doi: 10.7554/eLife.14158 (PMC4887207; doi:10.7554/eLife.14158)
Supplement: Related texts

# Article commentary: Decision letter

**DOI:** 10.7554/eLife.14158.023

**Affiliations:** University of California, Davis, United States

In the interests of transparency, eLife includes the editorial decision letter and accompanying author responses. A lightly edited version of the letter sent to the authors after peer review is shown, indicating the most substantive concerns; minor comments are not usually included.

Thank you for submitting your work entitled "Viral hijacking of a replicative helicase loader and its implications for helicase loading control and phage replication" for consideration by *eLife*. Your article has been reviewed by two peer reviewers, and the evaluation has been overseen by a Reviewing Editor and Richard Losick as the Senior Editor.

The reviewers have discussed the reviews with one another and the Reviewing Editor has drafted this decision to help you prepare a revised submission.

Drs. Hood and Berger describe an elegant structural and biochemical characterization of a phage-encoded replication inhibitor and its interaction with the host helicase loader. This is a concise and thoughtfully written manuscript that provides important structural and mechanistic insights. The work is of a very high technical quality, the manuscript is extremely well written, and it provides an accurate interpretation of the work. Most significantly, the authors solve a high resolution structure of the AAA+ ATPase domain of *S. aureus* DnaI (the first structures of this protein) in complex with an inhibitory phage encoded peptide 77ORF104 and the ATP analog, ADP•BeF_3_, along with an apo structure of the same DnaI domain by itself. Interpretation of the structural data is supported by biochemical data using mutant proteins and domain constructs. The structures are well-determined at high resolution. The authors propose the hypothesis that the inhibitor targets the helicase loader and, in doing so, modulates the conformation of the ISM, presumably altering higher order complex formation and thus accounting for the inhibitor's impact on the ATPase activity of the helicase loader.

Only one major question needs to be resolved:

1) While the structural work strongly supports the inhibitor-mediated ISM deformation and the inhibitor mediated attenuation of ATPase activity support the hypothesis of impaired oligomer formation, some direct proof that the inhibitor does alter the ability of DnaI to form higher order complexes is needed; e.g., simple gel filtration experiments could be performed to test this. It is important to know how the helicase and loader assemble in the absence of DNA, whether the phage peptide affects the assembly, and whether the putative phage loader also assembles similarly with the helicase. This has not been done with *S. aureus* proteins before. Have you analyzed these complexes by gel filtration? One should be able to mix DnaI and the helicase with or without the peptide and see a 6:6 complex by gel filtration/ion exchange. The same experiment needs to be done with the putative loader 77ORF013 to better substantiate your reasonable, but speculative, conclusion about 77ORF013.

Reviewer #1:

This is a concise and thoughtfully written manuscript that provides important structural and mechanistic insights into the involvement of the DnaI helicase loader in loading of the hexameric replicative helicase in *S. aureus*. Most significantly, the authors solve a high resolution structure of the AAA+ ATPase domain of *S. aureus* DnaI (the first structures of this protein) in complex with an inhibitory phage encoded peptide 77ORF104 and the ATP analog, ADP•BeF_3_, along with an apo structure of the same DnaI domain by itself. Interpretation of the structural data is supported by biochemical data using mutant proteins and domain constructs. The structures are well-determined at high resolution.

Interaction of DnaI with 77ORF104 is first confirmed, and its binding site on DnaI is shown to be the C-terminal AAA+ domain using pull-down and fluorescence polarization assays. These studies identified domain constructs suitable for crystallization. Comparison of the structure of the heterodimeric complex with the apo-DnaI structure and helicase loaders from other organisms revealed three features important for inhibition or potential regulation of helicase loading:

i) The binding site for 77ORF104 occludes the oligomerization interface for helical self-association of the AAA+ domains of DnaI thought to be required for helicase opening, based on the authors' previous structure of the DnaC helicase loader from *Aquifex aoelicus*. The assumption here is that helicase loading in Gram positive and negative organisms is similar, which is an unresolved question in the field (referring to earlier debates about ring-making vs. ring-breaking mechanisms, and the low-resolution Steitz X-ray structure of a Firmicute loader-helicase-primase complex).

ii) Comparison of the two new structures suggests the interactions of 77ORF104 leads to structural change in one helix in the ISM of DnaI (to a bent conformation); the ISM is a motif that drives helical assembly vs. ring assembly of the AAA+ domain of DnaA and DnaC. No argument is made that this rearrangement is not due to crystal contacts, and how it might affect helical assembly of DnaI (if it is required at all) is not explicitly discussed.

iii) A major site of interaction is in the C-terminus of 77ORF104, that forms a β strand that extends the sheet in DnaI (supported by deletion of this region abrogating the interaction). In the *G. kaustophilus* DnaI structure, this strand would occupy the same place as a strand from the interdomain linker (IDL) of DnaI. This suggests a regulatory role for the IDL in DnaI. What is not clear is why this interaction in the CTD of *S. aureus* DnaI (that contains the IDL as well as the AAA+ domain) apparently does not interfere with binding of 77ORF104.

These results raise the question of how the phage itself replicates when helicase loading is apparently prevented. It is suggested that the phage encodes an alternate loader (77ORF013). This protein is shown to interact with the helicase but not with 77ORF104, but it is not shown yet that it is actually an active helicase loader. A reasonable model is presented that accounts for all of these observations (with some speculation).

Reviewer #2:

Drs Berger and Hood describe an elegant structural and biochemical characterization of a phage-encoded replication inhibitor and its interaction with the host helicase loader. The work is of a very high technical quality, the manuscript is extremely well written and provides an accurate interpretation of the work. The authors propose the hypothesis that the inhibitor targets the helicase loader and, in doing so, modulates the conformation of the ISM, presumably altering higher order complex formation and thus accounting for the inhibitor's impact on the ATPase activity of the helicase loader.

While the structural work strongly supports the inhibitor-mediated ISM deformation and the inhibitor mediated attenuation of ATPase activity support the hypothesis of impaired oligomer formation, this referee would have liked to see some direct proof that the inhibitor does alter the ability of DnaI to form higher order complexes. As a bare minimum, some simple gel filtration experiments could be performed to test this.

It is intriguing that inhibitor binding does not affect the ability of the loader to interact with the helicase. The manuscript would be strengthened by inclusion of experiments that directly test the helicase loading activity of DnaI. Is the helicase brought to DNA but not released?

Finally, the authors identify a potential helicase-loader ortholog encoded by the phage and show that this protein can interact with the host helicase. These preliminary data are enticing. However, no further experiments are shown to test further the activity of this candidate loader. Does it have ATPase activity, is it modulated by ssDNA, can the protein load the helicase? Is the gene essential for the viral life-cycle? The manuscript requires these additional supporting experiments to test the functionality of 77ORF013.

# Reply: Author response

**DOI:** 10.7554/eLife.14158.024

Only one major question needs to be resolved:

*1) While the structural work strongly supports the inhibitor-mediated ISM deformation and the inhibitor mediated attenuation of ATPase activity support the hypothesis of impaired oligomer formation, some direct proof that the inhibitor does alter the ability of DnaI to form higher order complexes is needed; e.g., simple gel filtration experiments could be performed to test this. It is important to know how the helicase and loader assemble in the absence of DNA, whether the phage peptide affects the assembly, and whether the putative phage loader also assembles similarly with the helicase. This has not been done with S. aureus proteins before. Have you analyzed these complexes by gel filtration? One should be able to mix DnaI and the helicase with or without the peptide and see a 6:6 complex by gel filtration/ion exchange. The same experiment needs to be done with the putative loader 77ORF013 to better substantiate your reasonable, but speculative, conclusion about 77ORF013.*

We agree that these are important experiments. We initially considered using cross-linking and SDS-PAGE to examine self-self interactions between *Sa*DnaI protomers in the presence of both the *Sa*DnaC helicase and the phage 77 ORF104 protein. However, given the large number of possible interactions (loader-loader, loader-helicase, helicase-helicase), we realized that this experiment would likely be difficult to interpret cleanly (we do not have antibodies to any of the proteins in the study, which would have made such an approach more feasible). Instead, inspired by well-established studies of actin and microtubule self-assembly (1, 2), we have looked at loader interactions using a simple centrifugation assay. Based on studies that were initially not pertinent to the submitted manuscript (and hence not discussed), we have observed that, when chilled at elevated concentrations, *Sa*DnaI oligomerizes into visible microcrystals that can be sedimented after a brief centrifugation step. Were the phage inhibitor to disrupt DnaI-DnaI contacts, we reasoned that it should keep the loader in solution. As is now shown in Figure 4—figure supplement 1A of the revised manuscript, this is exactly what happens. In addition, we also looked at the behavior of *Sa*DnaI by dynamic light scattering. As can be seen and Figure 4—figure supplement 1B, the loader forms a very broad, polydisperse mixture of species with a large average molecular weight. By contrast, when measured in the presence of the inhibitor protein, a much smaller, less polydisperse species is formed (N.B. – the average molecular weight calculated for the dominant species in the DnaI-ORF104 mixture is larger than expected for a 1:1 complex, suggesting that inhibitor/loader complex formation is incomplete under the conditions tested here and/or that the N-terminal domain of DnaI might self-associate through unsatisfied, exposed surfaces that would normally be used for binding to the helicase). Collectively, these findings corroborate our other structural and biochemical data indicating that the phage inhibitor sterically blocks loader-loader interactions, and have been added to the revised manuscript (Figure 4—figure supplement 1).

Regarding the question as to the behavior of the *S. aureus* helicase/loader proteins and the phage inhibitor/loader homolog proteins by gel filtration, we have conducted such studies as well (Figure 4—figure supplement 2). These data indicate: 1) that the helicase *Sa*DnaC does not form a stable homo-hexameric ring but instead migrates as an apparent dimeric species (according to sizing standards run on the same column (not shown)),that the *Sa*DnaC helicase associates with both the *Sa*DnaI helicase loader and 2)the phage helicase loader homolog 77ORF013 (albeit with a degree of instability, as the bands for the helicase and DnaI/77ORF013 proteins do not perfectly overlap), and 3) that the phage 77 ORF104 inhibitor protein does not affect the migration of the *Sa*DnaI helicase loader, the phage 77ORF013 protein, or the *Sa*DnaC helicase.

Although at first glance the first two results may seem unsatisfying, they actually weigh in on a paradox in the field. In *B. subtilis*, helicase loading has been reported to occur not by a ring opening mechanism (as is thought to happen in *E. coli)* (3, 4), but rather by a DnaI-directed assembly process that additionally requires two co-chaperones not found in Gram-negative organisms, DnaB (which is unrelated to the *E. coli* DnaB helicase) and DnaD (5–7). Stable hexamers have been observed for Gram-positive DnaC helicases, but only in studies that analyze thermophilic proteins whose room-temperature behavior may differ from their mesophilic counterparts (e.g. *B. stearothermophilus* and *G. kaustophilus* (8–12)). It should be noted that dimers of DnaB-family helicases have been observed previously, wherein the dimer is stabilized by interactions between the N-terminal “collar” domains of the protein (13, 14).

Our findings, which show that *S. aureus* helicase/loader interactions form but are somewhat unstable, and that the DnaC helicase does not readily assemble a pre-formed hexamer, are in accord with observations reported for the *B. subtilis* system. We have added comments concerning how these data fit in with *B. subtilis* work to the Supplemental Information section of the revised paper. In the long term, we would like to attempt to reconstitute helicase loading in vitro for the *S. aureus* system, however, this will require also purifying *S. aureus* DnaA, DnaB and DnaD, and is beyond the scope of the present study.

Reviewer #1:

This is a concise and thoughtfully written manuscript that provides important structural and mechanistic insights into the involvement of the DnaI helicase loader in loading of the hexameric replicative helicase in S. aureus. Most significantly, the authors solve a high resolution structure of the AAA+ ATPase domain of S. aureus DnaI (the first structures of this protein) in complex with an inhibitory phage encoded peptide 77ORF104 and the ATP analog, ADP•BeF_3_, along with an apo structure of the same DnaI domain by itself. Interpretation of the structural data is supported by biochemical data using mutant proteins and domain constructs. The structures are well-determined at high resolution.

Interaction of DnaI with 77ORF104 is first confirmed, and its binding site on DnaI is shown to be the C-terminal AAA+ domain using pull-down and fluorescence polarization assays. These studies identified domain constructs suitable for crystallization. Comparison of the structure of the heterodimeric complex with the apo-DnaI structure and helicase loaders from other organisms revealed three features important for inhibition or potential regulation of helicase loading:

i) The binding site for 77ORF104 occludes the oligomerization interface for helical self-association of the AAA+ domains of DnaI thought to be required for helicase opening, based on the authors' previous structure of the DnaC helicase loader from Aquifex aoelicus. The assumption here is that helicase loading in Gram positive and negative organisms is similar, which is an unresolved question in the field (referring to earlier debates about ring-making vs. ring-breaking mechanisms, and the low-resolution Steitz X-ray structure of a Firmicute loader-helicase-primase complex).

Reviewer 1 brings up an excellent point here about the assumption “that helicase loading in Gram positive and negative organisms is similar”. Some aspects of this discussion are highlighted above in the response to the general comments. Based on our data and that in the literature, we feel it likely that some Gram-positive bacterial species, such as B. subtilis and Staphylococcus aureus, may use a ring assembly mechanism (rather than a ring-opening mechanism) that nonetheless also relies on AAA+ domain-dependent loader oligomerization (perhaps to help template the formation of helicase ring/spiral around DNA). We hope to study this question further in the future.

ii) Comparison of the two new structures suggests the interactions of 77ORF104 leads to structural change in one helix in the ISM of DnaI (to a bent conformation); the ISM is a motif that drives helical assembly vs. ring assembly of the AAA+ domain of DnaA and DnaC. No argument is made that this rearrangement is not due to crystal contacts, and how it might affect helical assembly of DnaI (if it is required at all) is not explicitly discussed.

Inspection of the crystal contacts in our structure indicates that the change in conformation of the ISM is not the result of lattice interactions. The conformational change also is incompatible with supporting productive DnaI-DnaI interactions capable for forming a proper bipartite active site. We have noted these points to the revised text.

iii) A major site of interaction is in the C-terminus of 77ORF104, that forms a β strand that extends the sheet in DnaI (supported by deletion of this region abrogating the interaction). In the G. kaustophilus DnaI structure, this strand would occupy the same place as a strand from the interdomain linker (IDL) of DnaI. This suggests a regulatory role for the IDL in DnaI. What is not clear is why this interaction in the CTD of S. aureus DnaI (that contains the IDL as well as the AAA+ domain) apparently does not interfere with binding of 77ORF104.

We believe there are two likely possibilities that can account for this result. One is that the conformational state of macromolecules is typically in an equilibrium. This equilibrium maybe biased to strongly favor the formation of one state, but nonetheless, other states can also be sampled. Hence, it is possible that 77ORF104 may simply take advantage of an inherent “breathing” event in *Sa*DnaI and bind at a point when the C-terminal AAA+ domain and the interdomain linker transiently separate. Alternatively, (or in addition), it is possible that the interdomain linker of DnaI uncouples from its resting place on the AAA+ domain when the loader’s N-terminal domain binds to the DnaC helicase, exposing the site for ORF104 binding. In the future, we would like to address this question using a combination of SAXS and fluorescence-based (FRET) measurements with appropriately labeled proteins.

Reviewer #2:

While the structural work strongly supports the inhibitor-mediated ISM deformation and the inhibitor mediated attenuation of ATPase activity support the hypothesis of impaired oligomer formation, this referee would have liked to see some direct proof that the inhibitor does alter the ability of DnaI to form higher order complexes. As a bare minimum, some simple gel filtration experiments could be performed to test this.

We agree that this is an important point. Please see above our new data and the response to the general comments section.

*It is intriguing that inhibitor binding does not affect the ability of the loader to interact with the helicase. The manuscript would be strengthened by inclusion of experiments that directly test the helicase loading activity of DnaI. Is the helicase brought to DNA but not released?*

We agree that this is an important point. Please see above our new data and the response to the general comments section.

Finally, the authors identify a potential helicase-loader ortholog encoded by the phage and show that this protein can interact with the host helicase. These preliminary data are enticing. However, no further experiments are shown to test further the activity of this candidate loader. Does it have ATPase activity, is it modulated by ssDNA, can the protein load the helicase? Is the gene essential for the viral life-cycle? The manuscript requires these additional supporting experiments to test the functionality of 77ORF013.

We agree that these are all excellent experiments; however, it is our feeling that properly carrying out the aggregate of the requested experiments – 1) ORF013 ATPase activity (+/-DNA, +/- the DnaC helicase), 2) ORF013 DNA binding (+/- ATP, +/- the DnaC helicase), 3) origin-dependent helicase loading (which requires identifying the phage origin of replication and identifying/purifying any associated initiator), and 4) essentiality (which requires making the appropriate phage mutants, packaging them in S. aureus with the appropriate helper phage, and looking for infectivity defects) – is beyond the scope of the present study. We hope the referee will agree.

References

1) J. Ziegelbauer et al., Transcription factor MIZ-1 is regulated via microtubule association. Mol. Cell. 8, 339–349 (2001).

2) J. Srivastava, D. Barber, Actin co-sedimentation assay; for the analysis of protein binding to F-actin. J. Vis. Exp., 2–3 (2008).

3) E. Arias-Palomo, V. L. O’Shea, I. V Hood, J. M. Berger, The bacterial DnaC helicase loader is a DnaB ring breaker. Cell. 153, 438–448 (2013).

4) M. J. Davey, M. O’Donnell, Replicative helicase loaders: ring breakers and ring makers. Curr Biol. 13, R594–6 (2003).

5) M. E. Rokop, J. M. Auchtung, A. D. Grossman, Control of DNA replication initiation by recruitment of an essential initiation protein to the membrane of Bacillus subtilis. Mol Microbiol. 52, 1757–1767 (2004).

6) W. K. Smits, A. I. Goranov, A. D. Grossman, Ordered association of helicase loader proteins with the Bacillus subtilis origin of replication in vivo. Mol Microbiol. 75, 452–461 (2010).

7) M. Velten et al., A two-protein strategy for the functional loading of a cellular replicative DNA helicase. Mol Cell. 11, 1009–1020 (2003).

8) S. Bailey, W. K. Eliason, T. A. Steitz, Structure of hexameric DnaB helicase and its complex with a domain of DnaG primase. Science (80-.). 318, 459–463 (2007).

9) Y. H. Lo et al., The crystal structure of a replicative hexameric helicase DnaC and its complex with single-stranded DNA. Nucleic Acids Res. 37, 804–814 (2009).

10) K. L. Tsai, Y. H. Lo, Y. J. Sun, C. D. Hsiao, thesis (2009).

11) P. Soultanas, A functional interaction between the putative primosomal protein DnaI and the main replicative DNA helicase DnaB in Bacillus. Nucleic Acids Res. 41, 5303–5320 (2002).

12) C. Ioannou, P. M. Schaeffer, N. E. Dixon, P. Soultanas, Helicase binding to DnaI exposes a cryptic DNA-binding site during helicase loading in Bacillus subtilis. Nucleic Acids Res. 34, 5247–5258 (2006).

13) S. Bailey, W. K. Eliason, T. A. Steitz, The crystal structure of the Thermus aquaticus DnaB helicase monomer. Nucleic Acids Res. 35, 4728–4736 (2007).

14) D. Fass, C. E. Bogden, J. M. Berger, Crystal structure of the N-terminal domain of the DnaB hexameric helicase. Structure. 7, 691–698 (1999).
